# Assessment of Social Risk Factors and Interest in Receiving Health Care–Based Social Assistance Among Adult Patients and Adult Caregivers of Pediatric Patients

**DOI:** 10.1001/jamanetworkopen.2020.21201

**Published:** 2020-10-16

**Authors:** Emilia H. De Marchis, Danielle Hessler, Caroline Fichtenberg, Eric W. Fleegler, Amy G. Huebschmann, Cheryl R. Clark, Alicia J. Cohen, Elena Byhoff, Mark J. Ommerborn, Nancy Adler, Laura M. Gottlieb

**Affiliations:** 1 Department of Family and Community Medicine, University of California, San Francisco, San Francisco; 2 Division of Emergency Medicine, Boston Children’s Hospital, Harvard Medical School, Boston, Massachusetts; 3 Division of General Internal Medicine and Center for Women’s Health Research, University of Colorado School of Medicine, Aurora; 4 Division of General Internal Medicine and Primary Care, Center for Community Health and Health Equity, Brigham and Women’s Hospital, Harvard Medical School, Boston, Massachusetts; 5 Providence VA Medical Center, Departments of Family Medicine and Health Services, Policy, and Practice, Brown University, Providence, Rhode Island; 6 Department of Medicine and Institute for Clinical Research and Health Policy Studies, Tufts Medical Center, Boston, Massachusetts; 7 Center for Community Health and Health Equity, Brigham and Women’s Hospital, Boston, Massachusetts; 8 Department of Psychiatry, University of California, San Francisco, San Francisco

## Abstract

**Importance:**

Health care organizations are increasingly incorporating social risk screening into patient care. Studies have reported wide variations in patients’ interest in receiving health care–based assistance for identified social risks. However, no study to date has examined the factors associated with patients’ interest in receiving assistance, including whether interest in receiving assistance varies based on specific patient demographic characteristics. Targeted research on this topic could improve the success of health care–based programs that offer social care services.

**Objective:**

To identify participant characteristics associated with interest in receiving health care–based social risk assistance.

**Design, Setting, and Participants:**

This cross-sectional study was conducted in 7 primary care clinics and 4 emergency departments in 9 US states between July 2, 2018, and February 13, 2019. A convenience sample of adult patients and adult caregivers of pediatric patients completed a screening survey that measured social risk factors and participants’ interest in receiving assistance for identified social risks. Participants were randomly selected to receive 1 of 2 versions of the survey, which differed based on the order in which questions about social risks and interest in receiving assistance were presented. Multivariable logistic regression analyses were used to evaluate the associations between covariates and participants’ interest in receiving assistance, stratified by social risk screening results. Data were analyzed from September 8, 2019, to July 30, 2020.

**Exposures:**

Social risk screening questions assessed risk factors comprising housing, food, transportation, utilities, and exposure to interpersonal violence. Additional questions assessed participants’ interest in receiving assistance and their perspectives on health care–based social risk screening.

**Main Outcomes and Measures:**

Participant interest in receiving health care–based social risk assistance.

**Results:**

A total of 1021 adult participants with complete survey responses were included in the analysis. Of those, 709 of 1004 participants (70.6%) were female, and 544 of 1007 participants (54.0%) were aged 18 to 44 years. Overall, 353 of 662 participants (53.3%) with positive screening results for 1 or more social risk factors were interested in receiving assistance, whereas 31 of 359 participants (8.6%) with negative screening results for all social risks were interested in receiving assistance. Participants with positive screening results for 1 or more social risk factors had a higher likelihood of being interested in receiving assistance if they answered the question about interest in receiving assistance before they answered the questions about social risk factors (adjusted odds ratio [aOR], 1.48; 95% CI, 1.05-2.07), had positive screening results for a higher number of social risk factors (aOR, 2.40; 95% CI, 1.68-3.42), reported lower household income levels (aOR, 7.78; 95% CI, 2.96-20.44), or self-identified as having non-Hispanic Black ancestry (aOR, 2.22; 95% CI, 1.37-3.60). Among those with negative screening results for all social risk factors, the interest in receiving assistance was higher if the participants reported lower household income levels (aOR, 12.38; 95% CI, 2.94-52.15), previous exposure to health care–based social risk screening (aOR, 2.35; 95% CI, 1.47-3.74), higher perceived appropriateness of social risk screening (aOR, 3.69; 95% CI, 1.08-12.55), or worse health status (aOR, 4.22; 95% CI, 1.09-16.31).

**Conclusions and Relevance:**

In this study, multiple factors were associated with participants’ interest in receiving social risk assistance. These findings may have implications for how and when social risk assistance is offered to patients. As the health care system’s role in addressing social risk factors evolves, an understanding of patients’ perspectives regarding screening and their interest in receiving assistance may be important to implementing patient-centered interventions.

## Key Points

**Question:**

Are specific social risk factors associated with patients’ interest in receiving health care–based social risk assistance?

**Findings:**

In this cross-sectional study of 1021 adult patients and adult caregivers of pediatric patients, 53% of participants who had positive screening results for social risks were interested in receiving social risk assistance compared with 9% of participants who had negative screening results. Among those with positive screening results, interest in receiving assistance was higher among participants who answered the question about their interest in receiving assistance before answering the questions about social risks and among participants who had positive results for multiple risks, reported lower household income, and self-identified as having non-Hispanic Black ancestry.

**Meaning:**

The study’s findings suggest that understanding patients’ perspectives regarding their interest in receiving social risk assistance may be an important consideration in the implementation of patient-centered social care interventions.

## Teaser

This cross-sectional study assesses social risk factors and examines characteristics associated with interest in receiving health care–based social risk assistance among adult patients and adult caregivers of pediatric patients.

## Introduction

Along with increasing recognition that patients’ social contexts may substantially impact their health, health care systems in the US are also increasing rates of screening patients for unmet social risk factors, such as food insecurity and housing instability,^1,2,3,4,5,6,7,8,9^ and are subsequently providing referrals to affiliated clinic or community social resources (ie, social risk assistance). This assistance may include the provision of food vouchers or help with the completion of applications for government benefits.^6,7,9,10,11,12,13,14,15,16,17,18^ As more organizations implement these types of social care initiatives across systems,^19^ variation has emerged regarding patients’ interest in receiving assistance from the health care system in response to identified social risks.^6,7,9,11,12,13,14,15,16,17,18,20^ For example, across previous studies, 25% to 94% of patients with positive screening results for food insecurity chose not to receive food assistance.^6,9,10,11,12,13,15,17^ In contrast, patients with negative screening results for social risks have sometimes expressed interest in receiving assistance.^11^ No study, to our knowledge, has examined the factors associated with patients’ interest in receiving assistance, including whether interest in receiving assistance varies based on specific demographic characteristics. Targeted research on this topic could improve the success of health care–based programs that offer social care services, with the goal of reducing health disparities and improving health outcomes.^20^

We aimed to identify factors associated with interest in receiving assistance among adult patients and adult caregivers of pediatric patients. We hypothesized that higher interest in receiving assistance would be associated with higher social risk burden,^21,22^ greater trust in clinicians,^13,23,24,25,26,27^ recruitment from safety-net settings, previous exposure to and perceived acceptability of social risk screening and social care activities,^23,27^ and fewer previous experiences with health care–based discrimination.^27,28,29,30^ We also hypothesized that the order in which survey questions were presented would be associated with participants’ interest in receiving assistance.

## Methods

We conducted a multisite cross-sectional study among a convenience sample of English- and Spanish-speaking adult patients and adult caregivers of pediatric patients from 7 primary care clinics and 4 emergency departments (EDs) in 9 US states between July 2, 2018, and February 13, 2019. To assess the association of the order in which survey questions were presented with participant responses, participants were randomly selected to receive 1 of 2 versions of the survey, which differed based on whether participants were asked about social risks before they were asked about their interest in receiving assistance or vice versa. The study was approved by the institutional review board of the University of California, San Francisco; 8 study sites (University of Arkansas, Boston Medical Center [Massachusetts], University of Chicago [Illinois], University of Colorado, Dartmouth College [New Hampshire], Hennepin Health [Minnesota], New York University [New York], and Brigham and Women’s Hospital [Massachusetts]) obtained additional site-specific institutional review board approval. All participants provided verbal informed consent. This study followed the Strengthening the Reporting of Observational Studies in Epidemiology (STROBE) reporting guideline.^31^

### Participants and Data Collection

Study participants were recruited from 11 clinical sites that provided health care services for a population in which at least 30% of patients were publicly insured or uninsured and that had not already implemented systematic social risk screening programs. The study sites included 4 family medicine clinics, 3 internal medicine clinics, 2 general EDs, and 2 pediatric EDs located in 9 states: Arkansas, California, Colorado, Illinois, Massachusetts, Minnesota, New Mexico, New York, and Vermont. Ten of 11 study sites recruited convenience samples of 100 adult patients or adult caregivers of pediatric patients; 1 internal medicine site recruited 50 participants because of limited time among staff members. Participants were eligible for inclusion if they (1) did not need immediate medical attention, (2) were 18 years or older, (3) were able to speak and read English and/or Spanish, (4) were comfortable using an electronic tablet, and (5) were able to provide informed consent. After providing verbal informed consent, participants completed a 32-item survey (eMethods in the Supplement) on their own (ie, without face-to-face or verbal interaction with survey administrators) using electronic tablets. Surveys took approximately 15 minutes to complete and were administered in private patient areas either before or after clinical visits. Participants were compensated $5.00 for their time. All patients and caregivers who were approached by research staff were offered information about local social services resources regardless of whether they decided to participate in the study.

Data were collected and managed using the REDCap online platform (REDCap Consortium and Vanderbilt University).^27,32^ Additional details regarding study site recruitment and eligibility criteria have been described in previous publications.^27,29^

### Outcome Measures

The primary outcome measure was participants’ interest in receiving assistance; this outcome was assessed through the following question: “Would you like to receive assistance with any of the following issues? (Check all that apply).” Response options included (1) housing; (2) food access; (3) medical or nonmedical transportation; (4) electric, gas, oil, or water utility services; (5) your safety, or violence in your household; or (6) none of these. Social risks were assessed using the Accountable Health Communities Health-Related Social Needs screening tool developed by the Center for Medicare and Medicaid Innovation^33^ and the Housing Stability Vital Sign developed by Children’s HealthWatch at Boston Medical Center.^34^ The Health-Related Social Needs 10-item screening tool assesses 5 social risk domains: (1) housing-associated social risks (ie, current instability or problems with quality of housing), (2) food insecurity, (3) transportation problems, (4) difficulty paying for utilities, and (5) exposure to interpersonal violence. The Housing Stability Vital Sign includes 3 questions that assess housing instability.^34^ Positive screening results were defined using the screening instruments’ criteria.^33^ Participants were considered to have positive results for a housing-associated social risk if they received positive results on either the Health-Related Social Needs or the Housing Stability Vital Sign tool.

The survey also included questions about participants’ perspectives on social risk screening (eg, whether they believed it was appropriate to screen for social risks in their health care setting), previous experiences with social risk screening and assistance (eg, whether they had been screened for social risks in a health care setting within the past year), previous experience with discrimination in a health care setting (eg, whether they had ever been treated differently by a physician or nurse because of their race, ethnicity, or socioeconomic status), trust in their clinician, and demographic characteristics (including age, sex, race, ethnicity, and household income). The full survey is available in eMethods in the Supplement.

Study sites were characterized by type of site (primary care vs ED). A site was considered a safety-net setting if at least 80% of patients who received services at the site were publicly insured or uninsured (based on self-reporting from each site).

### Statistical Analyses

The main outcome variable was participants’ interest in receiving assistance for any of the 5 social risk domains, dichotomized as any interest vs no interest. Because we assumed that interest in receiving assistance would differ based on whether participants received positive screening results for social risks, we stratified all analyses based on social risk endorsement (ie, positive results for any risk vs no risks). We used univariable analyses (2-sided Fisher exact χ^2^ tests) to assess bivariate associations with participants’ interest in receiving assistance. Associations between reported social risks and interest in receiving assistance for each risk domain were evaluated using Pearson correlation coefficients. Univariable and multivariable logistic regression analyses were used to identify factors independently associated with interest in receiving assistance. Robust SEs accounted for clustering by site. The multivariable analyses included variables that were associated with interest in receiving assistance at the *P* = .20 significance level in the univariable logistic regression analyses as well as variables that were hypothesized a priori to be associated with participants’ interest in receiving assistance.^35^ All independent variables were treated as categorical, with the exception of the burden of social risks, which was treated as ordinal (based on number of reported risks on a scale of 1-5).

For all regression models, we included participants with complete data after listwise deletion, with 1 exception for the household income variable, which had the most missing data (168 participants [16.5%]). For this variable, we included a separate category, defined as missing, to preserve the overall sample size and examine patterns of interest in receiving assistance among participants who did not respond to the income question.^36^ Because we assumed that missing data, such as household income and race/ethnicity (the variable with the second highest level of missing data, comprising 56 participants [5.5%]), were not missing at random, we did not conduct multiple imputations.^37,38,39,40^ Results were considered statistically significant at α < .05. We evaluated the impact of nonstratification based on social risk screening results through a pooled analysis. All analyses were conducted using Stata/SE software, version 15.1 (StataCorp). Data were analyzed from September 8, 2019, to July 30, 2020.

## Results

Overall, 1771 adult patients and adult caregivers of pediatric patients were invited to participate in the study (Figure). Among the 1054 participants (59.5%) who provided informed consent and responded to the survey, 1021 participants (96.9%) answered the question about interest in receiving assistance and were included in the final analysis. Most participants in the final sample were women (709 of 1004 participants [70.6%]) and aged 18 to 44 years (544 of 1007 participants [54.0%]) (Table 1).

**Figure.**
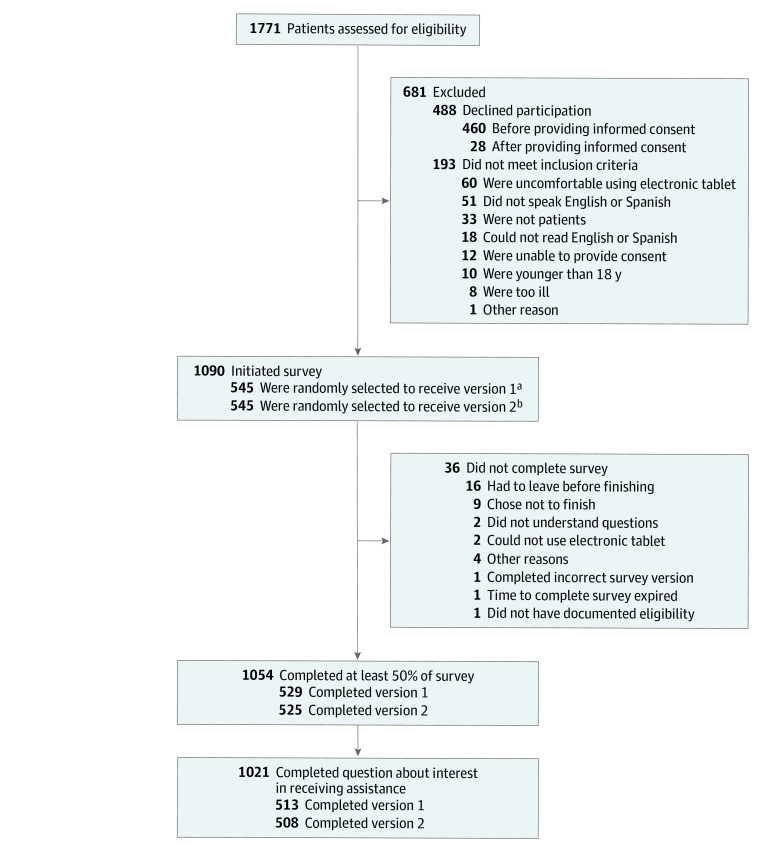
Flowchart of Participant Recruitment and Survey Completion ^a^Participants who received version 1 completed the social risk screening questions first. ^b^Participants who received version 2 completed the question regarding interest in receiving social risk assistance first.

**Table 1.** Participant Characteristics by Social Risk Screening Results and Interest in Receiving Assistance^a^

Characteristic	Participants, No./total No. (%)				
	With complete survey responses^b^	With positive screening results for ≥1 social risk factor^c^		With negative screening results for all social risk factors^d^	
		Interested in assistance	Not interested in assistance	Interested in assistance	Not interested in assistance
Participants	1021	353/662 (53.3)	309/662 (46.7)	31/359 (8.6)	328/359 (91.4)
**Participant characteristics**					
Age range, y					
18-44	544/1007 (54.0)	209/381 (54.9)	172/381 (45.1)	14/163 (8.6)	149/163 (91.4)
45-64	299/1007 (29.7)	105/194 (54.1)	89/194 (45.9)	10/105 (9.5)	95/105 (90.5)
≥65	164/1007 (16.3)	33/77 (42.9)	44/77 (57.1)	7/87 (8.0)	80/87 (92.0)
Sex					
Female	709/1004 (70.6)	251/466 (53.9)	215/466 (46.1)	17/243 (7.0)	226/243 (93.0)
Male	295/1004 (29.4)	96/184 (52.2)	88/184 (47.8)	13/111 (11.7)	98/111 (88.3)
Race/ethnicity					
Non-Hispanic White	359/965 (37.2)	68/185 (36.8)	117/185 (63.2)	10/174 (5.7)	164/174 (94.3)
Non-Hispanic Black	211/965 (21.9)	103/150 (68.7)	47/150 (31.3)	6/61 (9.8)	55/61 (90.2)
Hispanic	311/965 (32.2)	131/233 (56.2)	102/233 (43.8)	10/78 (12.8)	68/78 (87.2)
Non-Hispanic other race or multiple races	84/965 (8.7)	28/52 (53.8)	24/52 (46.2)	4/32 (12.5)	28/32 (87.5)
Preferred language					
English	848/1021 (83.1)	261/521 (50.1)	260/521 (49.9)	26/327 (8.0)	301/327 (92.0)
Spanish	173/1021 (16.9)	92/141 (65.2)	49/141 (34.8)	5/32 (15.6)	27/32 (84.4)
Educational level, y					
<12	181/1009 (17.9)	100/146 (68.5)	46/146 (31.5)	3/35 (8.6)	32/35 (91.4)
≥12	828/1009 (82.1)	248/508 (48.8)	260/508 (51.2)	28/320 (8.8)	292/320 (91.3)
Household income, $					
0-10 000	225/1021 (22.0)	142/195 (72.8)	53/195 (27.2)	6/30 (20.0)	24/30 (80.0)
10 001-25 000	189/1021 (18.5)	78/146 (53.4)	68/146 (46.6)	10/43 (23.3)	33/43 (76.7)
25 001-50 000	182/1021 (17.8)	61/126 (48.4)	65/126 (51.6)	4/56 (7.1)	52/56 (92.9)
50 001-75 000	80/1021 (7.8)	8/35 (22.9)	27/35 (77.1)	2/45 (4.4)	43/45 (95.6)
≥75 001	177/1021 (17.3)	5/56 (8.9)	51/56 (91.1)	3/121 (2.5)	118/121 (97.5)
Missing	168/1021 (16.5)	59/104 (56.7)	45/104 (43.3)	6/64 (9.4)	58/64 (90.6)
Self-reported health or caregiver-reported child’s health^41^					
Excellent, very good, or good	748/986 (75.9)	225/454 (49.6)	229/454 (50.4)	10/294 (6.8)	274/294 (93.2)
Fair or poor	238/986 (24.1)	112/182 (61.5)	70/182 (38.5)	10/56 (17.9)	46/56 (82.1)
Participant type					
Adult patient	793/1021 (77.7)	249/498 (50.0)	249/498 (50.0)	24/295 (8.14)	271/295 (91.9)
Adult caregiver of pediatric patient	228/1021 (22.3)	104/164 (63.4)	60/164 (36.6)	7/64 (10.9)	57/64 (89.1)
Trust in clinician^42^					
Complete (rating of 10)	504/981 (51.4)	163/313 (52.1)	150/313 (47.9)	13/191 (6.8)	178/191 (93.2)
High (rating of 8-9)	285/981 (29.1)	86/178 (48.3)	92/178 (51.7)	9/107 (8.4)	98/107 (91.6)
Medium to low (rating of 1-7)	192/981 (19.6)	86/144 (59.7)	58/144 (40.3)	7/48 (14.6)	41/48 (85.4)
Any previous experience with discrimination in a health care setting^43,44^					
Yes	274/1005 (27.3)	110/211 (52.1)	101/211 (47.9)	7/63 (11.1)	56/63 (88.9)
No	731/1005 (72.7)	235/440 (53.4)	205/440 (46.6)	22/291 (7.6)	269/291 (92.4)
Order of response to survey questions					
Answered questions about social risk factors first	513/1021 (50.2)	162/330 (49.1)	168/330 (50.9)	14/183 (7.7)	169/183 (92.3)
Answered question about interest in receiving assistance first	508/1021 (49.8)	191/332 (57.5)	141/332 (42.5)	17/176 (9.7)	159/176 (90.3)
**Social risk screening**^33,45^					
Risk factors, No.					
0	359/1021 (35.2)	0	0	31/359 (8.6)	328/359 (91.4)
1	257/1021 (25.2)	81/257 (31.5)	176/257 (68.5)	NA	NA
2	227/1021 (22.2)	128/227 (56.4)	99/227 (43.6)	NA	NA
3	135/1021 (13.2)	104/135 (77.0)	31/135 (23.0)	NA	NA
4	40/1021 (3.9)	37/40 (92.5)	3/40 (7.5)	NA	NA
5	3/1021 (0.3)	3/3 (100.0)	0	NA	NA
Any exposure to social risk screening in health care setting within past 12 mo					
Yes	313/1002 (31.2)	150/243 (61.7)	93/243 (38.3)	7/70 (10.0)	63/70 (90.0)
No	689/1002 (68.7)	194/409 (47.4)	215/409 (52.6)	22/279 (7.9)	258/280 (92.1)
Any social risk assistance in health care setting within past 12 mo					
Yes	182/1002 (18.2)	123/163 (75.5)	40/163 (24.5)	5/19 (26.3)	14/19 (73.7)
No	820/1002 (81.8)	219/487 (45.0)	268/487 (55.0)	25/333 (7.5)	308/333 (92.5)
Any discomfort with questions in any screening domain					
Yes	69/998 (6.9)	42/62 (67.7)	20/62 (32.3)	2/7 (28.6)	5/7 (71.4)
No	929/998 (93.1)	301/586 (51.4)	285/586 (48.6)	28/343 (8.2)	315/343 (91.8)
Perceptions of appropriateness of health care–based social risk screening					
Very appropriate or somewhat appropriate	782/982 (79.6)	276/515 (53.6)	239/515 (46.4)	26/267 (9.7)	241/267 (90.3)
Neither, very inappropriate, or somewhat inappropriate	200/982 (20.4)	60/122 (49.2)	62/122 (50.8)	3/78 (3.8)	75/78 (96.2)
Comfort with integrating social risk data into EHR					
Completely comfortable or somewhat comfortable	632/982 (64.4)	229/403 (56.8)	174/403 (43.2)	18/229 (7.9)	211/229 (92.1)
Neither, completely uncomfortable, or somewhat uncomfortable	350/982 (35.6)	107/234 (45.7)	127/234 (54.3)	11/116 (9.5)	105/116 (90.5)
Health care setting					
Primary care	628/1021 (61.5)	199/398 (50.0)	199/398 (50.0)	15/230 (6.5)	215/230 (93.5)
Emergency department	393/1021 (38.5)	154/264 (58.3)	110/264 (41.7)	16/129 (12.4)	113/129 (87.6)
Individual study sites					
1	101/1021 (9.9)	56/75 (74.7)	19/75 (25.3)	5/26 (19.2)	21/26 (80.8)
2	100/1021 (9.8)	24/59 (40.7)	35/59 (59.3)	6/41 (14.6)	35/41 (85.4)
3	98/1021 (9.6)	58/81 (71.6)	23/81 (28.4)	4/17 (23.5)	13/17 (76.5)
4	98/1021 (9.6)	34/58 (58.6)	24/58 (41.4)	3/40 (7.5)	37/40 (92.5)
5	95/1021 (9.3)	40/75 (53.3)	35/75 (46.7)	1/20 (5.0)	19/20 (95.0)
6	97/1021 (9.5)	38/66 (57.6)	28/66 (42.4)	3/31 (9.7)	28/31 (90.3)
7	99/1021 (9.7)	23/50 (46.0)	27/50 (54.0)	2/49 (4.1)	47/49 (95.9)
8	89/1021 (8.7)	17/41 (41.5)	24/41 (58.5)	3/48 (6.3)	45/48 (93.8)
9	96/1021 (9.4)	35/71 (49.3)	36/71 (50.7)	1/25 (4.0)	24/25 (96.0)
10	100/1021 (9.8)	21/67 (31.3)	46/67 (68.7)	0	33/33 (100.0)
11	48/1021 (4.7)	7/19 (36.8)	12/19 (63.2)	3/29 (10.3)	26/29 (89.7)
Participants with public insurance or no insurance, %					
<80	726/1021 (71.1)	204/435 (46.9)	231/435 (53.1)	21/292 (7.2)	270/291 (92.8)
≥80	295/1021 (28.9)	149/227 (65.6)	78/227 (34.4)	10/68 (14.7)	58/68 (85.3)

Abbreviations: EHR, electronic health record; NA, not applicable.

^a^ A total of 33 of 1054 participants (3.1%) completed at least 50% of the survey (the study’s threshold for completion) but did not answer the question about interest in receiving assistance. These participants were more likely to be Hispanic, to speak Spanish, to report better health, and to have been recruited from a primary care setting compared with other participants (eTable 1 in the Supplement).

^b^ Denominators are based on the total number of participants with complete responses for each specific characteristic.

^c^ Denominators are based on the total number of participants with positive screening results for 1 or more risk factors.

^d^ Denominators are based on the total number of participants with negative screening results for all risk factors.

### Interest in Receiving Assistance

A total of 384 participants (37.6% of the study sample) expressed interest in receiving assistance with at least 1 social risk. Interest in receiving assistance was higher among the 662 participants who had a positive screening result for at least 1 social risk (353 participants [53.3%] were interested in receiving assistance) than among the 359 participants who had negative screening results (31 participants [8.6%] were interested in receiving assistance). Across the 11 study sites, interest in receiving assistance among those who received a positive screening result for 1 or more social risk factors ranged from 21 of 67 participants (31.3%) to 56 of 75 participants (74.7%) (*P* < .001). Table 1 shows the unadjusted associations between independent variables and interest in receiving assistance, stratified by social risk status.

Among 1021 participants, the interest in risk-specific assistance was highest for housing (232 participants [22.7%]), followed by food (167 participants [16.4%]), transportation (121 participants [11.8%]), utilities (114 participants [11.2%]), and safety (19 participants [1.9%]). Interest in receiving social risk–specific assistance among those with a positive screening result for any single risk factor was consistent across most social risk domains, ranging from 144 of 417 participants (34.5%) who were interested in receiving food assistance to 79 of 200 participants (39.5%) who were interested in receiving transportation assistance. The sole exception was interpersonal violence, for which only 2 of 18 participants (11.1%) who had a positive screening result were interested in receiving assistance. Interest in receiving risk-specific assistance among those with a negative screening result for any single risk factor ranged from 16 of 966 participants (1.7%) who were interested in receiving assistance for interpersonal violence to 39 of 494 participants (7.9%) who were interested in receiving assistance for housing (Table 2). Bivariate associations with risk-specific interest in receiving assistance are available in eTable 2 through eTable 6 in the Supplement. Interest in receiving assistance with food was associated with interest in receiving assistance with transportation (Pearson *r* = 0.37; *P* < .001), housing (Pearson *r* = 0.37; *P* < .001), and utilities (Pearson *r* = 0.32; *P* < .001).

**Table 2.** Participant Interest in Receiving Risk-Specific Assistance, Stratified by Response to Specific Social Risk Screening Questions

Specified risk domain	Participants, No./total No. (%)							*P* value^d^
	With complete survey responses, No.	With positive screening results			With negative screening results			
		Total^a^	Interested in assistance^b^	Not interested in assistance^b^	Total^a^	Interested in assistance^c^	Not interested in assistance^c^	
Housing	1021	527/1021 (51.6)	193/527 (36.6)	334/527 (63.4)	494/1021 (48.4)	39/494 (7.9)	455/494 (92.1)	<.001
Food	1011	417/1011 (41.2)	144/417 (34.5)	273/417 (65.5)	594/1011 (58.8)	22/594 (3.7)	572/594 (96.3)	<.001
Utilities	1001	129/1001 (12.9)	45/129 (34.9)	84/129 (65.1)	872/1001 (87.1)	66/872 (7.6)	806/872 (92.4)	<.001
Transportation	998	200/998 (20.0)	79/200 (39.5)	121/200 (60.5)	798/998 (80.0)	39/798 (4.9)	759/798 (95.1)	<.001
Interpersonal violence	984	18/984 (1.8)	2/18 (11.1)	16/18 (88.9)	966/984 (98.2)	16/966 (1.7)	950/966 (98.3)	.04
Any risks	1021	662/1021 (64.8)	353/662 (53.3)	309/662 (46.7)	359/1021 (35.2)	31/359 (8.6)	328/359 (91.4)	<.001

^a^ Denominators are based on the total number of participants with complete responses (column 1).

^b^ Denominators are based on the total number of participants with a positive screening result for each specified risk domain (column 2).

^c^ Denominators are based on the total number of participants with a negative screening result for each specified risk domain (column 5).

^d^ *P* value for Fisher exact χ^2^ analysis evaluating differences in participants’ levels of interest in receiving assistance by social risk screening result.

### Multivariable and Pooled Analyses

Participants with a positive screening result for 1 or more social risk factors had a significantly higher likelihood of being interested in receiving assistance if they were asked the question about interest in receiving assistance before they were asked the questions about social risk (adjusted odds ratio [aOR], 1.48; 95% CI, 1.05-2.07; *P* = .02), had positive screening results for a higher number of social risks (aOR, 2.40; 95% CI, 1.68-3.42; *P* < .001), reported lower household income (aOR, 7.78; 95% CI, 2.96-20.44; *P* < .001), or self-identified as having non-Hispanic Black ancestry (aOR, 2.22; 95% CI, 1.37-3.60; *P* = .001). Participants who received negative screening results had a significantly higher likelihood of being interested in assistance if they reported lower household income (aOR, 12.38; 95% CI, 2.94-52.15; *P* = .001), reported previous exposure to health care–based social risk screening (aOR, 2.35; 95% CI, 1.47-3.74; *P* < .001), reported higher perceived appropriateness of health care–based social risk screening (aOR, 3.69; 95% CI, 1.08-12.55; *P* = .04), reported worse health (aOR, 4.22; 95% CI, 1.09-16.31; *P* = .04), or were recruited from an ED setting (aOR, 4.27; 95% CI, 1.59-11.45; *P* = .004).

Regardless of social risk screening results, participants’ interest in receiving assistance was not associated with trust in clinicians, recruitment from a safety-net setting, or previous experiences of health care–based discrimination. Stratified unadjusted and adjusted ORs for interest in receiving assistance are shown in Table 3, and stratified adjusted predicted probabilities for interest in receiving assistance are shown in Table 4. Comparison of independent variables for the 851 participants included in the multivariable models and the 170 participants excluded from the analysis owing to missing data are available in eTable 7 in the Supplement.

**Table 3.** Unadjusted and Adjusted Associations Between Social Risk Factors and Interest in Receiving Assistance, Stratified by Response to Social Risk Screening Questions^a^

Variable	Participants with positive screening results for ≥1 social risk factor (n = 550)				Participants with negative screening results for all social risk factors (n = 301)			
	Unadjusted OR (95% CI)	*P* value	Adjusted OR (95% CI)	*P* value	Unadjusted OR (95% CI)	*P* value	Adjusted OR (95% CI)	*P* value
**Participant characteristics**								
Age range, y								
18-44	1 [Reference]	NA	1 [Reference]	NA	1 [Reference]	NA	1 [Reference]	NA
45-64	0.79 (0.50-1.26)	.32	1.03 (0.67-1.58)	.91	1.22 (0.48-3.09)	.68	3.88 (0.67-22.35)	.13
≥65	0.61 (0.37-1.00)	.05	1.20 (0.70-2.05)	.51	0.92 (0.40-2.11)	.85	2.10 (0.42-10.43)	.36
Race/ethnicity								
Non-Hispanic White	1 [Reference]	NA	1 [Reference]	NA	1 [Reference]	NA	1 [Reference]	NA
Non-Hispanic Black	3.78 (2.00-7.17)	<.001	2.22 (1.37-3.60)	.001	1.94 (1.08-3.49)	.03	0.57 (0.18-1.88)	.36
Hispanic	1.95 (1.34-2.85)	.001	0.74 (0.29-1.91)	.53	2.99 (1.02-8.81)	.05	1.51 (0.27-8.51)	.64
Non-Hispanic other race or multiple races	2.05 (1.10-3.80)	.02	1.36 (0.76-2.44)	.30	3.39 (0.80-14.28)	.10	1.43 (0.27-7.58)	.67
Preferred language								
English	1 [Reference]	NA	1 [Reference]	NA	1 [Reference]	NA	1 [Reference]	NA
Spanish	1.55 (0.90-2.66)	.12	2.20 (0.97-4.97)	.06	2.45 (1.24-4.82)	.01	2.58 (0.49-4.70)	.17
Educational level, y								
<12	1.84 (1.20-2.82)	.005	1.46 (0.81-2.65)	.21	1.01 (0.29-3.54)	.99	0.21 (0.02-2.18)	.19
≥12	1 [Reference]	NA	1 [Reference]	NA	1 [Reference]	NA	1 [Reference]	NA
Household income, $								
Missing	10.74 (3.89-29.70)	<.001	3.93 (1.06-14.57)	.04	2.67 (0.49-14.61)	.26	2.23 (0.22-22.06)	.49
0-10 000	34.13 (17.2-67.58)	<.001	7.78 (2.96-20.44)	<.001	10.40 (3.91-27.65)	<.001	12.38 (2.94-52.15)	.001
10 001-25 000	12.16 (4.70-31.43)	<.001	3.90 (1.19-12.75)	.03	10.76 (5.06-22.86)	<.001	11.48 (2.44-53.94)	.002
25 001-50 000	12.16 (6.06-24.43)	<.001	5.45 (2.49-11.96)	<.001	2.36 (0.36-15.34)	.37	3.40 (0.41-28.37)	.26
50 001-75 000	3.62 (1.00-13.01)	.05	2.37 (0.59-9.51)	.22	1.78 (0.41-7.74)	.44	1.37 (0.28-6.77)	.70
≥75 001	1 [Reference]	NA	1 [Reference]	NA	1 [Reference]	NA	1 [Reference]	NA
Self-reported health or caregiver-reported child’s health								
Excellent, very good, or good	1 [Reference]	NA	1 [Reference]	NA	1 [Reference]	NA	1 [Reference]	NA
Fair or poor	1.66 (1.03-2.69)	.04	1.50 (0.92-2.46)	.11	2.95 (1.53-5.71)	.001	4.22 (1.09-16.31)	.04
Participant type								
Adult patient	1 [Reference]	NA	1 [Reference]	NA	1 [Reference]	NA	1 [Reference]	NA
Adult caregiver of pediatric patient	2.03 (1.12-3.68)	.02	1.35 (0.52-3.50)	.53	1.51 (0.53-4.25)	.44	2.60 (0.66-10.32)	.17
Trust in clinician								
Complete (rating of 10)	0.76 (0.48-1.19)	.23	1.13 (0.64-1.99)	.66	0.43 (0.12-1.55)	.20	0.56 (0.08-4.11)	.57
High (rating of 8-9)	0.72 (0.44-1.17)	.19	1.31 (0.77-2.23)	.31	0.49 (0.20-1.17)	.11	0.70 (0.18-2.69)	.60
Medium to low (rating of 1-7)	1 [Reference]	NA	1 [Reference]	NA	1 [Reference]	NA	1 [Reference]	NA
Any previous experience with discrimination in a health care setting								
Yes	0.98 (0.71-1.35)	.89	0.71 (0.48-1.05)	.09	1.58 (0.57-4.38)	.38	1.37 (0.25-7.55)	.71
No	1 [Reference]	NA	1 [Reference]	NA	1 [Reference]	NA	1 [Reference]	NA
Order of response to survey questions								
Answered questions about social risk factors first	1 [Reference]	NA	1 [Reference]	NA	1 [Reference]	NA	1 [Reference]	NA
Answered question about interest in receiving assistance first	1.22 (0.85-1.76)	.27	1.48 (1.05-2.07)	.02	1.76 (0.66-4.67)	.26	1.52 (0.49-4.70)	.47
**Social risk screening**								
No. of risk factors^b^	2.86 (2.15-3.82)	<.001	2.40 (1.68-3.42)	<.001	NA	NA	NA	NA
Any exposure to social risk screening in health care setting within past 12 mo								
Yes	1.78 (1.01-3.14)	.05	1.42 (0.90-2.26)	.14	1.26 (0.50-3.16)	.63	2.35 (1.47-3.74)	<.001
No	1 [Reference]	NA	1 [Reference]	NA	1 [Reference]	NA	1 [Reference]	NA
Any social risk assistance in health care setting within past 12 mo								
Yes	3.98 (2.57-6.15)	<.001	1.66 (0.97-2.82)	.06	4.36 (0.99-19.29)	.05	2.00 (0.42-9.45)	.38
No	1 [Reference]	NA	1 [Reference]	NA	1 [Reference]	NA	1 [Reference]	NA
Any discomfort with questions in any screening domain								
Yes	2.54 (1.26-5.10)	.009	1.43 (0.54-3.75)	.47	3.63 (0.81-16.20)	.09	5.75 (0.64-51.58)	.12
No	1 [Reference]	NA	1 [Reference]	NA	1 [Reference]	NA	1 [Reference]	NA
Perceptions of appropriateness of health care–based social risk screening								
Very appropriate or somewhat appropriate	1.04 (0.73-1.50)	.81	0.74 (0.44-1.25)	.26	2.19 (0.74-6.43)	.16	3.69 (1.08-12.55)	.04
Neither, very inappropriate, or somewhat inappropriate	1 [Reference]	NA	1 [Reference]	NA	1 [Reference]	NA	1 [Reference]	NA
Comfort with integrating social risk data into EHR								
Completely comfortable or somewhat comfortable	1.49 (0.92-2.42)	.11	1.48 (0.90-2.45)	.13	0.70 (0.29-1.69)	.43	0.42 (0.08-2.22)	.31
Neither, completely uncomfortable, or somewhat uncomfortable	1 [Reference]	NA	1 [Reference]	NA	1 [Reference]	NA	1 [Reference]	NA
Health care setting								
Primary care	1 [Reference]	NA	1 [Reference]	NA	1 [Reference]	NA	1 [Reference]	NA
Emergency department	1.47 (0.72-3.02)	.30	1.39 (0.69-2.79)	.35	2.55 (1.03-6.32)	.04	4.27 (1.59-11.45)	.004
Participants with public insurance or no insurance, %								
<80	1 [Reference]	NA	1 [Reference]	NA	1 [Reference]	NA	1 [Reference]	NA
≥80	2.23 (1.15-4.34)	.02	1.62 (0.73-3.57)	.24	2.50 (0.91-6.86)	.07	1.91 (0.74-4.90)	.18

Abbreviations: EHR, electronic health record; NA, not applicable; OR, odds ratio.

^a^ The table shows all variables included in the adjusted analyses. Models were run as logistic regressions with clustered SEs that were categorized by clinical site. Sample size presented is the size after listwise deletion. Among participants interested in assistance, those excluded from the regression model were older and more likely to be Hispanic, to speak Spanish, to have lower educational levels and household income, and to report discomfort with answering the social risk screening questions (eTable 7 in the Supplement). Among participants not interested in assistance, those excluded from the regression model were older and more likely to be adult patients (vs adult caregivers), to have lower educational levels, to have been recruited from a primary care setting, and to report that health care–based social risk screening was less appropriate (eTable 7 in the Supplement).

^b^ The number of risk factors ranged from 0 to 5.

**Table 4.** Adjusted Predicted Probabilities of Interest in Receiving Assistance for Social Risks, Stratified by Response to Social Risk Screening Questions

Variable	Adjusted probability, % (95% CI)	
	Participants with positive screening results for ≥1 social risk factor (n = 550)	Participants with negative screening results for all social risk factors (n = 301)
**Participant characteristics**		
Age range, y		
18-44	51.4 (47.1 to 55.6)	6.1 (2.9 to 9.2)
45-64	51.8 (44.7 to 58.9)	15.1 (4.6 to 25.5)
≥65	54.4 (45.1 to 63.7)	10.2 (2.7 to 17.8)
Race/ethnicity		
Non-Hispanic White	49.8 (40.7 to 58.9)	8.4 (3.5 to 13.3)
Non-Hispanic Black	63.4 (58.1 to 68.8)	5.6 (1.9 to 9.2)
Hispanic	44.5 (33.9 to 55.1)	11.1 (1.1 to 21.1)
Non-Hispanic other race or multiple races	55.2 (42.6 to 67.7)	10.7 (1.5 to 19.9)
Preferred language		
English	49.0 (44.4 to 53.6)	8.0 (6.1 to 9.9)
Spanish	62.3 (50.7 to 74.0)	15.1 (4.3 to 25.9)
Educational level, y		
<12	57.1 (48.2 to 66.0)	3.0 (−1.5 to 7.5)
≥12	50.6 (45.8 to 55.4)	10.0 (6.9 to 13.1)
Household income, $		
Missing	48.9 (38.2 to 59.7)	6.2 (−2.9 to 15.3)
0-10 000	61.8 (54.2 to 69.5)	21.4 (8.9 to 33.9)
10 001-25 000	48.8 (38.0 to 59.5)	20.4 (4.8 to 36.1)
25 001-50 000	55.2 (48.1 to 62.2)	8.7 (−3.4 to 20.7)
50 001-75 000	39.6 (24.7 to 54.4)	4.1 (−1.5 to 9.7)
≥75 001	25.5 (9.9 to 41.0)	3.1 (1.0 to 5.2)
Self-reported health or caregiver-reported child's health		
Excellent, very good, or good	49.8 (45.2 to 54.5)	6.8 (5.1 to 8.7)
Fair or poor	56.8 (49.3 to 64.4)	17.8 (7.3 to 28.4)
Participant type		
Adult patient	50.5 (43.7 to 57.2)	7.4 (5.4 to 9.4)
Adult caregiver of pediatric patient	55.7 (44.3 to 67.1)	13.9 (5.3 to 22.6)
Trust in clinician		
Complete (rating of 10)	51.6 (46.2 to 57.0)	7.6 (2.8 to 12.5)
High (rating of 8-9)	54.1 (48.9 to 59.4)	8.9 (5.1 to 12.7)
Medium to low (rating of 1-7)	49.5 (41.3 to 57.6)	11.4 (2.2 to 20.5)
Any previous experience with discrimination in a health care setting		
Yes	47.8 (41.2 to 54.4)	10.3 (0.4 to 20.2)
No	53.7 (49.5 to 57.9)	8.3 (5.8 to 10.7)
Order of response to survey questions		
Answered questions about social risk factors first	48.4 (41.7 to 55.2)	7.3 (3.2 to 11.5)
Answered question about interest in receiving assistance first	55.1 (53.0 to 57.3)	9.8 (6.5 to 13.2)
**Social risk screening**		
Risk factors, No.		
1	36.2 (27.5 to 44.8)	NA
2	53.2 (48.6 to 57.9)	NA
3	69.6 (63.5 to 75.6)	NA
4	82.3 (73.9 to 90.8)	NA
5	90.8 (82.9 to 98.7)	NA
Any exposure to social risk screening in health care setting within past 12 mo		
Yes	49.8 (45.6 to 54.0)	13.7 (9.3 to 18.2)
No	55.9 (48.0 to 63.7)	7.7 (6.2 to 9.3)
Any social risk assistance in health care setting within past 12 mo		
Yes	58.7 (51.3 to 66.0)	13.1 (2.5 to 23.7)
No	49.8 (44.8 to 54.9)	8.2 (6.0 to 10.4)
Any discomfort with questions in any screening domain		
Yes	57.4 (41.0 to 73.9)	24.5 (−2.5 to 51.4)
No	51.4 (47.4 to 55.4)	8.4 (6.7 to 10.1)
Perceptions of appropriateness of health care–based social risk screening		
Very appropriate or somewhat appropriate	50.8 (45.7 to 56.0)	10.4 (7.8 to 12.9)
Neither, very inappropriate, or somewhat inappropriate	55.9 (49.8 to 62.0)	3.8 (0.4 to 7.3)
Comfort with integrating social risk data in EHR		
Completely comfortable or somewhat comfortable	54.4 (49.0 to 59.8)	7.1 (3.5 to 10.7)
Neither, completely uncomfortable, or somewhat uncomfortable	47.6 (41.3 to 54.0)	12.7 (5.0 to 20.3)
Health care setting		
Primary care	49.5 (43.4 to 55.4)	5.1 (2.4 to 7.7)
Emergency department	55.2 (46.4 to 64.0)	14.2 (9.9 to 18.5)
Participants with public insurance or no insurance, %		
<80	49.2 (45.0 to 53.2)	7.6 (5.2 to 10.0)
≥80	57.4 (45.3 to 69.6)	11.9 (6.0 to 17.7)

Abbreviations: EHR, electronic health record; NA, not applicable.

In the pooled analysis of the full sample, variables associated with greater interest in receiving assistance included having positive screening results for a greater number of risks, answering the question about interest in receiving assistance before answering the questions about social risk, and reporting lower income, worse health, non-Hispanic Black ancestry, and Spanish (vs English) language preference (eTable 8 in the Supplement). Language preference was the only variable that was not associated with interest in receiving assistance in the stratified models. In the pooled model of participants without social risks, the 3 variables that were associated with interest in receiving assistance in the stratified models (previous exposure to health care–based social risk screening, higher perceived appropriateness of health care–based social risk screening, and recruitment from an ED setting) were no longer associated.

## Discussion

In this multisite cross-sectional study of factors associated with patients’ interest in receiving social risk assistance in health care settings, we found that 53.3% of participants with a positive screening result for a social risk were interested in receiving assistance, and 8.6% of those with negative screening results were also interested in receiving assistance. Among participants with a positive screening result for at least 1 social risk, interest in receiving assistance was higher in those who answered the question about interest in receiving assistance before answering the questions about specific social risks; interest in receiving assistance was also higher in those who had a higher cumulative number of social risks, reported lower household income, and self-identified as having non-Hispanic Black ancestry. Among those with negative screening results for all 5 social risks, interest in receiving assistance was also associated with lower household income. In this group, interest in receiving assistance was also associated with worse self-rated health, previous exposure to health care–based social risk screening, higher perceived appropriateness of health care–based social risk screening, and recruitment from an ED setting; interest in receiving assistance was not associated with race/ethnicity, or the order in which questions were presented. Participants’ interest in receiving assistance, regardless of social risk screening results, was not associated with trust in clinicians, recruitment from a safety-net setting, or previous experiences of health care–based discrimination.

The proportion of patients interested in social risk assistance has varied in previous studies.^6,7,9,10,11,12,13,14,15,16,17,18,20^ In the present study, we found that 53.3% of those with positive screening results were interested in receiving assistance, although results varied by study site. Our finding that 8.6% of participants were interested in assistance despite receiving negative screening results is consistent with previous research.^10,11,20^ In the context of gaps in the data regarding the psychometric properties of social risk screening tools,^46^ the interest in receiving assistance among those with negative screening results might indicate that assistance initiatives should not be limited to those with positive screening results. Among those with negative screening results, higher interest in receiving assistance was associated with previous exposure to and higher acceptability of social risk screening, which suggests that as social care activities become more common in health care settings, interest in receiving assistance may increase.^47^

To our knowledge, this study is the first to examine the association between the order in which survey questions are presented and participant-reported interest in receiving social risk assistance. We found that interest in receiving assistance may increase when interest is assessed before specific social risks. It is possible that reviewing the risks first may have implications for individuals’ perspectives about whether they qualify for assistance, while asking about assistance first could help to convey that the reason for asking about risks is to provide assistance. Future studies are warranted to explore how the order in which survey questions are presented is associated with respondents’ interest in receiving assistance or the benefits of screening in comparison with more universal provision of assistance.^11,48,49,50^

Contrary to our a priori hypotheses, interest in receiving assistance was not associated with trust in clinicians, recruitment from a safety-net setting, or previous experiences with health care–based discrimination. Trust in clinicians was high at baseline, which may account for the lack of association between trust in clinicians and interest in receiving assistance in our sample. A previous analysis of this sample found that previous experience with discrimination was associated with lower perceived acceptability of social risk screening.^27^ In the present analyses, previous experience with discrimination was associated with lower interest in receiving assistance among those reporting social risk but higher interest in receiving assistance among those not reporting social risk; however, neither finding was statistically significant.

We found similar levels of interest in receiving assistance for housing, food, utilities, and transportation among participants with positive screening results for those risk factors. Lower levels of interest in receiving assistance were observed among participants with positive screening results for interpersonal safety. These findings, along with the low rates of reported interpersonal violence, are consistent with the literature^12,51,52,53,54^ and highlight the challenges in providing intervention services for individuals experiencing interpersonal violence.

### Limitations

This study has several limitations. First, as a cross-sectional study, the analyses cannot indicate causation. Second, the study excluded patients and caregivers who did not speak or read English or Spanish. Third, although a diverse set of study sites was included, the sites are not representative of all health care settings. Fourth, the survey question about participants’ interest in receiving assistance was asked theoretically, as part of a research study rather than an intervention. Fifth, our analyses are limited by the variables included in the survey. Additional reasons that those with positive screening results may not be interested in receiving assistance include the receipt of false-positive results,^46^ the perception among patients that they are already familiar with available resources, and the ways in which assistance is offered.^6,7,8,13,14,15,20,29,55^ Among those with negative screening results, the additional reasons that patients may be interested in receiving assistance are similar and include the low sensitivity of the screening tools,^46^ concerns among patients about the consequences of endorsing social needs,^23^ and the likelihood that social needs are associated with one another.^56,57^ Sixth, the study relied on participant-reported information that could not be validated. Despite these limitations, this study has a number of strengths, including its randomized survey design and its diverse study sites and participant sample.

## Conclusions

In this multisite cross-sectional study, we found multiple factors that were associated with participants’ interest in receiving social risk assistance in health care settings. For participants with positive screening results for social risks, these factors included answering survey questions about assistance before answering questions about social risks, receiving positive results for a higher number of social risks, and reporting lower household income. Interest in receiving assistance was also expressed by those with negative screening results for social risks, especially those with lower income and worse self-reported health. These findings may have implications for how and when social risk assistance is offered to patients. For example, health care organizations might opt to first offer or describe assistive services before conducting social risk screening. As the health care system’s role in addressing social risk factors evolves, more work is needed to understand patients’ perspectives on social risk screening, and patients’ interest in receiving assistance can be used to augment efforts to implement patient-centered social care interventions.

## Notes

Supplement. **eMethods.** Study Survey Tool **eTable 1.** Differences in Participant and Health Center Characteristics Between Study Sample and Respondents Excluded From Sample **eTable 2.** Participant Characteristics by Interest in Housing Assistance and Housing-Related Social Risk Screening Results **eTable 3.** Participant Characteristics by Interest in Food Assistance and Food Insecurity Screening Results **eTable 4.** Participant Characteristics by Interest in Utilities Assistance and Utilities Screening Results **eTable 5.** Participant Characteristics by Interest in Transportation Assistance and Transportation Problems Screening Results **eTable 6.** Participant Characteristics by Interest in Safety Assistance and Interpersonal Violence Screening Results **eTable 7.** Differences in Participant Characteristics, Stratified by Social Risk Screening Results, Between Respondents Included in Multivariable Model and Respondents Excluded Owing to Missingness **eTable 8.** Pooled Analyses: Unadjusted and Adjusted Associations Between Interest in Assistance Among All Participants
