# Supplementary material for: Assessment of Social Risk Factors and Interest in Receiving Health Care–Based Social Assistance Among Adult Patients and Adult Caregivers of Pediatric Patients
Source: JAMA Netw Open. 2020 Oct 16;3(10):e2021201. doi: 10.1001/jamanetworkopen.2020.21201 (PMC7568201; doi:10.1001/jamanetworkopen.2020.21201)
Supplement: Supplement. — eMethods. Study Survey Tool eTable 1. Differences in Participant and Health Center Characteristics Between Study Sample and Respondents Excluded From Sample eTable 2. Participant Characteristics by Interest in Housing Assistance and Housing-Related Social Risk Screening Results eTable 3. Participant Characteristics by Interest in Food Assistance and Food Insecurity Screening Results eTable 4. Participant Characteristics by Interest in Utilities Assistance and Utilities Screening Results eTable 5. Participant Characteristics by Interest in Transportation Assistance and Transportation Problems Screening Results eTable 6. Participant Characteristics by Interest in Safety Assistance and Interpersonal Violence Screening Results eTable 7. Differences in Participant Characteristics, Stratified by Social Risk Screening Results, Between Respondents Included in Multivariable Model and Respondents Excluded Owing to Missingness eTable 8. Pooled Analyses: Unadjusted and Adjusted Associations Between Interest in Assistance Among All Participants [file jamanetwopen-e2021201-s001.pdf]

Supplementary Online Content

De Marchis EH, Hessler D, Fichtenberg C, et al. Assessment of social risk factors and interest in receiving health care–based social assistance among adult patients and adult caregivers of pediatric patients. *JAMA Netw Open*. 2020;3(10):e2021201. doi:10.1001/jamanetworkopen.2020.21201

eMethods. Study Survey Tool

eTable 1. Differences in Participant and Health Center Characteristics Between Study Sample and Respondents Excluded From Sample

eTable 2. Participant Characteristics by Interest in Housing Assistance and Housing-Related Social Risk Screening Results

eTable 3. Participant Characteristics by Interest in Food Assistance and Food Insecurity Screening Results

eTable 4. Participant Characteristics by Interest in Utilities Assistance and Utilities Screening Results

eTable 5. Participant Characteristics by Interest in Transportation Assistance and Transportation Problems Screening Results

eTable 6. Participant Characteristics by Interest in Safety Assistance and Interpersonal Violence Screening Results

eTable 7. Differences in Participant Characteristics, Stratified by Social Risk Screening Results, Between Respondents Included in Multivariable Model and Respondents Excluded Owing to Missingness

eTable 8. Pooled Analyses: Unadjusted and Adjusted Associations Between Interest in Assistance Among Participants With 1 or More Social Risk Factor

This supplementary material has been provided by the authors to give readers additional information about their work.

© 2020 De Marchis EH et al. *JAMA Network Open.*

eMethods. Study Survey Tool

The SIREN Accountable Health Communities screening tool study

RA marks one of the following:
___ *Participant is receiving care today*
___ *Participant is the adult caregiver of a child receiving care today*

We appreciate your participation in this survey. Your responses will help us understand more about how to develop programs that can help respond to the needs of our patients.

AHC Screening instrument

Housing Stability and Conditions

1. What is your housing situation today?
   a) I have a steady place to live
   b) I have a place to live today, but I am worried about losing it in the future
   c) I do not have a steady place to live (I am temporarily staying with others, in a hotel, in a shelter, living outside on the street, on a beach, in a car, abandoned building, bus or train station, or in a park)
   d) I prefer not to answer[a]

2. Think about the place you live. Do you have problems with any of the following? (Check all that apply)
   a) Pests such as bugs, ants, or mice
   b) Mold
   c) Lead paint or pipes
   d) Lack of heat
   e) Oven or stove not working
   f) Smoke detectors missing or not working
   g) Water leaks
   h) None of the above
   i) I prefer not to answer

Food Security

Some people have made the following statements about their food situation. Please answer whether the statements were OFTEN, SOMETIMES, or NEVER true for you and your household in the last 12 months.

3. Within the past 12 months, you worried that your food would run out before you got money to buy more.
   a) Often true
   b) Sometimes true
   c) Never true
   d) I prefer not to answer

© 2020 De Marchis EH et al. *JAMA Network Open*.

4. Within the past 12 months, the food you bought just didn't last and you didn't have money to get more.
   a) Often true
   b) Sometimes true
   c) Never true
   d) I prefer not to answer

Transportation Needs

5. In the past 12 months, has lack of reliable transportation kept you from medical appointments, meetings, work or from getting things needed for daily living?
   a) Yes
   b) No
   c) I prefer not to answer

Utility Needs

6. In the past 12 months has the electric, gas, oil, or water company threatened to shut off services in your home?
   a) Yes
   b) No
   c) Already shut off
   d) I prefer not to answer

Interpersonal Safety

Because violence and abuse happens to a lot of people and affects their health we are asking the following questions.

7. How often does anyone, including family and friends, physically hurt you?
   a) Never
   b) Rarely
   c) Sometimes
   d) Fairly often
   e) Frequently
   f) I prefer not to answer

8. How often does anyone, including family and friends, insult or talk down to you?
   a) Never
   b) Rarely
   c) Sometimes
   d) Fairly often
   e) Frequently
   f) I prefer not to answer
9. How often does anyone, including family and friends, threaten you with harm?

© 2020 De Marchis EH et al. *JAMA Network Open*.

a) Never
b) Rarely
c) Sometimes
d) Fairly often
e) Frequently
f) I prefer not to answer

10. How often does anyone, including family and friends, scream or curse at you?
a) Never
b) Rarely
c) Sometimes
d) Fairly often
e) Frequently
f) I prefer not to answer

A score of 11 or more when the numerical values for answers to questions 7-10 are added shows that the person might not be safe.

Additional housing questions

11. In the past 12 months, was there a time when you were not able to pay the mortgage or rent on time?
a) Yes
b) No

12. In the past 12 months, how many times have you moved where you were living? (*drop down*) *(Answer is # of moves. Positive screen if answer is 2 or more moves in the last 12 months)*

13. At any time in the past 12 months, were you homeless or living in shelter (including now)?
a) Yes
b) No

Desire for help with needs

14. Would you like to receive assistance with any of the issues below: (check all that apply)[b]
a) Housing
b) Food access
c) Medical or non-medical transportation
d) Electric, gas, oil, or water utility services
e) Your safety, or violence in your household
f) None of these

Patient Acceptability of Social Screening

15. In the last 12 months, not including today, have you been asked about any of the following in any health care setting: (check all that apply)

© 2020 De Marchis EH et al. *JAMA Network Open*.

a) Yes, housing
b) Yes, food access
c) Yes, medical or non-medical transportation
d) Yes, electric, gas, oil, or water utility services
e) Yes, your safety, or violence in your household
f) No, none of these

16. In the last 12 months, not including today, have you received assistance from anyone in any health care setting related to: (check all that apply)
a) Housing
b) Food access
c) Medical or non-medical transportation
d) Electric, gas, oil, or water utility services
e) Your safety, or violence in your household
f) None of these

17a. *Branch if primary care*: How long have you or your family been receiving care at this clinic?
a) Less than 1 year
b) 1 to less than 3 years
c) 3 to less than 5 years
d) 5 years of more

17b. *Branch if adult ED patient*: Is there a doctor or place that you usually go if you are sick or need advice about your health?
a) Yes
b) No

17c. *Branch if adult caregiver of pediatric ED patient*: Is there a doctor or place that you usually go if your child is sick or you need advice about your child's health?
c) Yes
d) No

18. Do you think it is appropriate to be asked these questions about your social and economic needs at [BRANCH: "this clinic" OR "this emergency department"]?
a) Very appropriate
b) Somewhat appropriate
c) Neither appropriate nor inappropriate
d) Somewhat inappropriate
e) Very inappropriate

19. Please check if you felt uncomfortable today being asked any of the questions about: (check all that apply)
a) Housing
b) Food access
c) Medical or non-medical transportation

© 2020 De Marchis EH et al. *JAMA Network Open*.

d) Electric, gas, oil, or water utility services
e) Your safety, or violence in your household
f) None of these

20. In general, where do you think people should be asked questions about their social and economic needs? Check all that apply.
a) Emergency Department
b) Primary Care/Pediatrician's Office
c) Other health care settings (e.g. specialty clinics, hospital)
d) Non-health care settings (e.g. school, community center)
e) None of these places

21. How frequently do you think it is appropriate to be asked these questions about social and economic needs?
(*Branch based on location*)

i. In a primary care outpatient clinic visit:
    a. every time I receive care
    b. once every 6 months
    c. once a year
    d. every 2 years
    e. every 5 years
    f. never

ii. In the emergency room/urgent care:
    a. every time I receive care
    b. once every 6 months
    c. once a year
    d. every 2 years
    e. every 5 years
    f. never

22. Would you be comfortable having these kinds of needs included in your health records (also known as your medical record or chart)?
a) Completely comfortable
b) Somewhat comfortable
c) Neither comfortable nor uncomfortable
d) Somewhat uncomfortable
e) Completely uncomfortable

Self-rated health

23a. In general, would you say your health is…(select one)
a) Excellent
b) Very good
c) Good

d) Fair
e) Poor
f) Don't know/refused to answer

BRANCH: If patient is a child, ***survey will branch*** *to include parent-reported child health question below*

23b. In general, would you say your child's health is… (select one)

a) Excellent
b) Very good
c) Good
d) Fair
e) Poor
f) Don't know/refused to answer

Patient trust in their provider

24. *BRANCH if patient in clinic*: How much do you trust your health care provider(s) at this clinic?
BRANCH if caregiver of pediatric patient in clinic: How much do you trust your child's health care provider(s) at this clinic?
BRANCH if ED: How much do you trust the health care providers at this emergency department?

1	2	3	4	5	6	7	8	9	10
Not at all									Completely

Demographics

25. What is your age?

a) 18 to 24
b) 25 to 34
c) 35 to 44
d) 45 to 54
e) 55 to 64
f) 65 to 74
g) 75 or older

26. What is the highest grade (or year) of school you completed? (select one)

Elementary School	High School	College	Graduate School
01_____	09_____	13_____	17_____
02_____	10_____	14_____	18_____
03_____	11_____	15_____	19_____
04_____	12_____	16_____	20+____
05_____			

© 2020 De Marchis EH et al. *JAMA Network Open*.

06_____
07_____
08_____

27. How do you describe your gender?
a) Male
b) Female
c) Trans male/Trans man
d) Trans female/Trans woman
e) Gender-queer/Gender non-conforming
f) Different identity
g) Prefer not to answer

28. What is your race? (mark all that apply)
a) White
b) Black or African American
c) American Indian or Alaska Native
d) Asian Indian
e) Chinese
f) Filipino
g) Japanese
h) Korean
i) Vietnamese
j) Native Hawaiian
k) Guamanian or Chamorro
l) Samoan
m) Other Pacific Islander (specify) ____________
n) Other Asian (specify)______________
o) Some other race (specify) _____________

29. Are you of Hispanic or Latino origin?
a) No, not Hispanic, Latino or Spanish origin
b) Yes, Mexican, Mexican American, Chicano
c) Yes, Puerto Rican
d) Yes, Cuban
e) Yes, another Hispanic, Latino, or Spanish origin _________________

30a. Which of the following categories best describes your total combined household income for the past 12 months?
a) 0 - $5,000
b) $5,001 - $10,000
c) $10,001 - $15,000
d) $15,001 - $20,000
e) $20,001 - $25,000
f) $25,001 - $30,000
g) $30,001 - $35,000

© 2020 De Marchis EH et al. *JAMA Network Open*.

h) $35,001 - $40,000
i) $40,001 - $50,000
j) $50,001 - $75,000
k) $75,001 - $100,000
l) $100,001 - $150,000
m) $150,000 +
n) Don't know
o) Would rather not say

30b. How many people (kids and adults) are currently dependent on this income and living in your household, including yourself? (*drop down*)
(Answer is # of people in household dependent on income)

30c. Of these people, how many are 0-17 years old? (*drop down*)
(Answer is # of people in household 0-17 years old)

31. When getting health care, have you ever had any of the following things happen to you because of your race, ethnicity, or socioeconomic status?

a) Felt like a doctor or nurse was not listening to what you were saying
   Drop down Yes/No
b) Treated you with less respect than other people
   Drop down Yes/No
c) Received poorer services than other people
   Drop down Yes/No
d) Treated with less courtesy than other people
   Drop down Yes/No
e) Had a doctor or nurse act as if he or she was better than you
   Drop down Yes/No
f) Had a doctor or nurse act as if he or she thinks you were not smart
   Drop down Yes/No
g) Had a doctor or nurse act as if he or she was afraid of you
   Drop down Yes/No

32. Finally, we would like to understand where you see yourself in relation to others in the United States.

Think of the ladder below as representing where people stand in the United States.
At the top of the ladder (10) are the people who are the best off—those who have the most money, the most education, and the most respected jobs.
At the bottom (1) are the people who are the worst off—who have the least money, least education, and the least respected jobs or no job. The higher up you are on this ladder, the closer you are to the people at the very top.

Where would you put yourself on this ladder?

© 2020 De Marchis EH et al. *JAMA Network Open*.

Please respond with the number (1-10) where you think you stand at this time in your life, relative to other people in the United States.

Answer 1-10

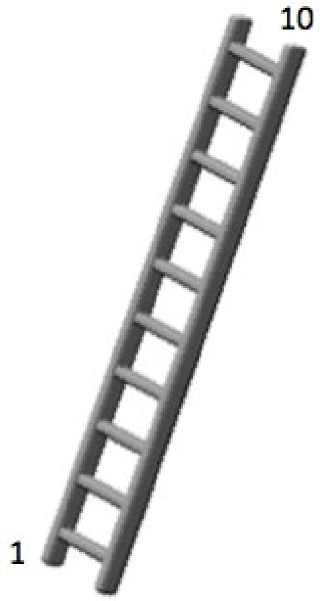

[a]50% of participants were randomized to complete a version of survey with the “I prefer not to answer” option for questions #1-10.
[b]50% of participants were randomized such that question #14 appeared before question #1 (before the social risk screening questions, instead of after).

© 2020 De Marchis EH et al. *JAMA Network Open*.

eTable 1. Differences in Participant and Health Center Characteristics Between Study Sample (N = 1021) and Respondents Excluded From Sample (N = 33)

Variable (N[a])	Completed at least 50% of survey (N=1054)		
	Answered question about interest in assistance (included in study sample)	Skipped question about interest in assistance (excluded from study sample)	Fisher's exact p value
	(N=1021, 97%) No. (%)	(N=33, 3%) No. (%)	
Participant characteristics			
Age (years) (1038)			
18-44	544 (54%)	17 (55%)	0.62
45-64	299 (30%)	11 (35%)	
≥65	164 (16%)	3 (10%)	
Sex (1034)			
Female	709 (71%)	19 (63%)	0.42
Male	295 (29%)	11 (37%)	
Race/Ethnicity (992)			
Non-Hispanic White	359 (37%)	8 (30%)	0.001
Non-Hispanic Black	211 (22%)	1 (4%)	
Hispanic	311 (32%)	18 (67%)	
Non-Hispanic Other/Multiple Races	84 (9%)	0 (0%)	
Education (N=1042)			
<12 years	181 (18%)	8 (24%)	0.36
≥12 years	828 (82%)	25 (76%)	

© 2020 De Marchis EH et al. *JAMA Network Open*.

eTable 1. Continued			
Variable (N[a])	Completed at least 50% of survey (N=1054)		Fisher's exact p value
	Answered question about interest in assistance (included in study sample)	Skipped question about interest in assistance (excluded from study sample)	
	(N=1021, 97%) No. (%)	(N=33, 3%) No. (%)	
Income (N=1054)			
Missing	168 (16.5%)	7 (21.2%)	0.86
$0-10,000	225 (22.0%)	5 (15.2%)	
$10,001-25,000	189 (18.5%)	5 (15.2%)	
$25,001-50,000	182 (17.8%)	7 (21.2%)	
$50,001-75,000	80 (7.8%)	2 (6.1%)	
>$75,001	177 (17.3%)	7 (21.2%)	
Preferred Language (1054)			
English	848 (83%)	21 (64%)	0.008
Spanish	173 (17%)	12 (36%)	
Self-Reported Health or Child's Health (1019)			
Excellent/Very good/Good	748 (76%)	31 (94%)	0.01
Fair/Poor	238 (24%)	2 (6%)	
Participant type (1054)			
Adult patient	793 (78%)	26 (79%)	1.00
Adult caregiver of pediatric patient	228 (22%)	7 (21%)	

© 2020 De Marchis EH et al. *JAMA Network Open*.

eTable 1. Continued			
Variable (N[a])	Completed at least 50% of survey (N=1054)		
	Answered question about interest in assistance (included in study sample)	Skipped question about interest in assistance (excluded from study sample)	Fisher's exact p value
	(N=1021, 97%) No. (%)	(N=33, 3%) No. (%)	
Social Risk Screening			
Housing instability (1054)			
Yes	527 (52%)	11 (33%)	0.05
No	494 (48%)	22 (67%)	
Food insecurity (1044)			
Yes	417 (41%)	11 (33%)	0.47
No	594 (59%)	22 (67%)	
Utilities problems (1033)			
Yes	129 (13%)	0 (0%)	0.03
No	872 (87%)	32 (100%)	
Transportation problems (1031)			
Yes	200 (20%)	2 (6%)	0.05
No	798 (80%)	31 (94%)	
Safety concern (1014)			
Yes	18 (2%)	0 (0%)	1.00
No	966 (98%)	30 (100%)	

© 2020 De Marchis EH et al. *JAMA Network Open*.

eTable 1. Continued			
Variable (N[a])	Completed at least 50% of survey (N=1054)		
	Answered question about interest in assistance (included in study sample)	Skipped question about interest in assistance (excluded from study sample)	Fisher's exact p value
	(N=1021, 97%) No. (%)	(N=33, 3%) No. (%)	
Overall social risk (1054)			
No risk factors	359 (35.2%)	18 (54.6%)	0.14
1	257 (25.2%)	7 (21.2%)	
2	227 (22.2%)	7 (21.2%)	
3	135 (13.2%)	1 (3.03%)	
4-5	43 (4.21%)	0 (0.00%)	
Any prior social risk screening exposure in a health care setting in the past 12 months (1002)			
Yes	313 (31%)	7 (25%)	0.54
No	689 (69%)	21 (75%)	
Any prior social risk assistance from health care setting in the past 12 months (1030)			
Yes	182 (18%)		0.45
No	820 (82%)	25 (89%)	
Any discomfort with questions in any screening domains (1026)			
Yes	69 (7%)	2 (7%)	1.00
No	929 (93%)	26 (93%)	
Interest in any form of assistance (1021)			
Yes	384 (37.6%)	-	N/A
No	637 (62.4%)	-	

© 2020 De Marchis EH et al. *JAMA Network Open*.

eTable 1. Continued			
Variable (N[a])	Completed at least 50% of survey (N=1054)		
	Answered question about interest in assistance (included in study sample)	Skipped question about interest in assistance (excluded from study sample)	Fisher's exact p value
	(N=1021, 97%) No. (%)	(N=33, 3%) No. (%)	
Trust in clinician (1012)			
Complete (10)	504 (51%)	19 (61%)	0.61
High (8-9)	285 (29%)	7 (23%)	
Medium-Low (1-7)	192 (20%)	5 (16%)	
Appropriateness of health care-based social risk screening (1014)			
Very/somewhat appropriate	782 (80%)	24 (75%)	0.51
Neither; Very/somewhat inappropriate	200 (20%)	8 (25%)	
Comfort with integrating social risk data in EHR (1014)			
Completely/somewhat comfortable	632 (64%)	19 (59%)	0.58
Neither/completely/somewhat uncomfortable	350 (36%)	13 (41%)	
Any experience prior discrimination within health care (1038)			
Yes	274 (27%)	11 (33%)	0.43
No	731 (73%)	22 (67%)	
Health care setting characteristics (1054)			
Primary Care	628 (62%)	26 (79%)	0.05
Emergency Department	393 (38%)	7 (21%)	
Percentage patient population publicly-insured or uninsured (1054)			
<80%	726 (71%)	25 (76%)	
≥80%	295 (29%)	8 (24%)	0.70

© 2020 De Marchis EH et al. *JAMA Network Open*.

[a]Number of participants with complete responses for each variable

© 2020 De Marchis EH et al. *JAMA Network Open*.

eTable 2. Participant Characteristics by Interest in Housing Assistance and Housing-Related Social Risk Screening Results (N = 1021)

Variable	Total	Screened positive for housing-related social risk (N=527, 51.6%)		Fisher's exact p value	Screened negative for housing-related social risk (N=494, 43.4%)		Fisher's exact p value
		Interested in housing assistance (N=193, 36.6%) No. (%)	Not interested in housing assistance (N=334, 63.4%) No. (%)		Interested in housing assistance (N=39, 7.9%) No. (%)	Not interested in housing assistance (N=455, 92.1%) No. (%)	
Participant characteristics							
Age (years)							
18-44	544	122 (39.5)	187 (60.5)	0.07	23 (9.8)	212 (90.2)	0.02
45-64	299	54 (35.1)	100 (64.9)		14 (9.7)	131 (90.3)	
≥65	164	13 (23.6)	42 (76.4)		2 (1.8)	107 (98.2)	
Sex							
Female	709	134 (36.9)	229 (63.1)	0.84	26 (7.5)	320 (92.5)	0.58
Male	295	55 (35.7)	99 (64.3)		13 (9.2)	128 (90.8)	
Race/Ethnicity							
Non-Hispanic White	365	35 (23.2)	116 (76.8)	<0.001	7 (3.4)	207 (96.6)	0.003
Non-Hispanic Black	211	54 (46.2)	63 (53.9)		12 (12.8)	82 (87.2)	
Hispanic	311	76 (41.8)	106 (58.2)		16 (12.4)	113 (87.6)	
Non-Hispanic Other/Multiple Races	84	14 (33.3)	28 (66.7)		3 (7.1)	39 (92.9)	
Preferred Language							
English	848	142 (33.9)	27 (66.1)	0.01	31 (7.2)	398 (92.8)	0.21
Spanish	173	51 (47.2)	57 (52.8)		8 (12.3)	57 (87.7)	
Education							
<12 years	181	50 (47.6)	55 (52.4)	0.009	10 (13.2)	66 (86.8)	0.06
≥12 years	828	140 (33.7)	276 (66.4)		28 (6.8)	384 (93.2)	

© 2020 De Marchis EH et al. *JAMA Network Open*.

eTable 2. Continued							
Variable	Total	Screened positive for housing-related social risk (N=527, 51.6%)		Fisher's exact p value	Screened negative for housing-related social risk (N=494, 43.4%)		Fisher's exact p value
		Interested in housing assistance (N=193, 36.6%) No. (%)	Not interested in housing assistance (N=334, 63.4%) No. (%)		Interested in housing assistance (N=39, 7.9%) No. (%)	Interested in housing assistance (N=455, 92.1%) No. (%)	
Income							
Missing	168	30 (36.6%)	52 (63.4%)	<0.001	9 (10.5%)	77 (89.5%)	<0.001
$0-$10,000	225	85 (53.8%)	73 (46.2%)		12 (17.9%)	55 (82.1%)	
$10,001-$25,000	189	36 (33.6%)	71 (66.4%)		11 (13.4%)	71 (86.6%)	
$25,001-$50,000	182	34 (33.7%)	67 (66.3%)		5 (6.2%)	76 (93.8%)	
$50,001-$75,000	80	6 (19.4%)	25 (80.6%)		0 (0.0%)	49 (100.0%)	
≥$75001	177	2 (4.2%)	46 (95.8%)		2 (1.6%)	127 (98.4%)	
Self-Reported Health or Child's Health							
Excellent/Very good/Good	748	116 (32.1)	245 (67.9)	0.008	31 (8.0)	356 (92.0)	1.00
Fair/Poor	238	66 (44.9)	81 (55.1)		7 (7.7)	84 (92.3)	
Participant type							
Adult patient	793	140 (35.8)	251 (64.2)	0.54	27 (6.7)	375 (93.3)	0.05
Adult caregiver of pediatric patient	228	53 (39.0)	83 (61.0)		12 (13.0)	80 (87.0)	
Trust in clinician							
Complete (10)	504	79 (32.0)	168 (68.0)		20 (7.8)	237 (92.2)	
High (8-9)	285	50 (34.5)	95 (65.5)		9 (6.4)	131 (93.6)	
Medium-Low (1-7)	192	53 (46.9)	60 (53.1)	0.02	9 (11.4)	70 (88.6)	0.40

© 2020 De Marchis EH et al. *JAMA Network Open*.

eTable 2. Continued							
Variable	Total	Screened positive for housing-related social risk		Fisher's exact p value	Screened negative for housing-related social risk		Fisher's exact p value
		(N=527, 51.6%)			(N=494, 43.4%)		
		Interested in housing assistance	Not interested in housing assistance		Interested in housing assistance	Interested in housing assistance	
		(N=193, 36.6%) No. (%)	(N=334, 63.4%) No. (%)		(N=39, 7.9%) No. (%)	(N=455, 92.1%) No. (%)	
Any experience prior discrimination within health care							
Yes	274	64 (37.4)	125 (36.1)	0.77	5 (4.8)	98 (95.2)	0.30
No	731	125 (36.1)	221 (63.9)		33 (8.6)	352 (91.4)	
Ordering of questions about social risk screening and question about interest in assistance							
Answered questions about social risk factors first	513	91 (35.0)	169 (65.0)	0.47	16 (6.3)	237 (93.7)	0.24
Answered question about interest in assistance first	508	165 (61.8)	102 (38.2)		23 (9.5)	218 (90.5)	
Social Risk Screening							
Number of risk factors							
0	359	-	-	<0.001	15 (4.2)	344 (95.8)	<0.001
1	257	30 (19.0)	128 (81.0)		18 (18.2)	81 (81.8)	
2	227	70 (36.1)	124 (63.9)		6 (18.2)	27 (81.8)	
3	135	62 (47.0)	70 (53.0)		0 (0.0)	3 (100.0)	
4	40	29 (72.5)	11 (27.5)		-	-	
5	3	2 (66.7)	1 (33.3)		-	-	
Any prior social risk screening exposure in a health care setting in the past 12 months							
Yes	313	95 (46.8)	108 (53.2)	<0.001	17 (15.5)	93 (84.5)	0.003
No	689	95 (29.9)	223 (70.0)		22 (5.9)	349 (94.1)	

© 2020 De Marchis EH et al. *JAMA Network Open*.

eTable 2. Continued							
Variable	Total	Screened positive for housing-related social risk		Fisher's exact p value	Screened negative for housing-related social risk		Fisher's exact p value
		(N=527, 51.6%)			(N=494, 43.4%)		
		Interested in housing assistance	Not interested in housing assistance		Interested in housing assistance	Interested in housing assistance	
		(N=193, 36.6%) No. (%)	(N=334, 63.4%) No. (%)		(N=39, 7.9%) No. (%)	(N=455, 92.1%) No. (%)	
Any prior social risk assistance from health care setting in the past 12 months							
Yes	182	75 (53.6)	65 (46.4)	<0.001	9 (21.4)	33 (78.6)	0.004
No	820	113 (29.9)	265 (70.1)		30 (6.8)	412 (93.2)	
Any discomfort with questions in any screening domains							
Yes	69	23 (42.6)	31 (57.4)	0.37	2 (13.3)	13 (86.7)	0.35
No	929	167 (36.0)	297 (64.0)		37 (8.0)	428 (92.0)	
Appropriateness of health care-based social risk screening							
Very/somewhat appropriate	782	152 (36.8)	261 (63.2)	0.41	35 (9.5)	334 (90.5)	0.03
Neither/Very/ somewhat inappropriate	200	30 (31.6)	65 (68.4)		3 (2.9)	102 (97.1)	
Comfort with integrating social risk data in EHR							
Completely/somewhat comfortable	637	127 (39.2)	197 (60.8)	0.04	29 (9.3)	284 (90.7)	0.35
Neither/completely/ somewhat uncomfortable	269	55 (29.9)	129 (70.1)		4 (4.7)	81 (95.3)	

© 2020 De Marchis EH et al. *JAMA Network Open*.

eTable 2. Continued							
Variable	Total	Screened positive for housing-related social risk		Fisher's exact p value	Screened negative for housing-related social risk		Fisher's exact p value
		(N=527, 51.6%)			(N=494, 43.4%)		
		Interested in housing assistance	Not interested in housing assistance		Interested in housing assistance	Interested in housing assistance	
		(N=193, 36.6%) No. (%)	(N=334, 63.4%) No. (%)		(N=39, 7.9%) No. (%)	(N=455, 92.1%) No. (%)	
Health care setting characteristics							
Primary Care	628	114 (36.7)	197 (63.3)	1.00	19 (6.0)	298 (94.0)	0.05
Emergency Department	393	79 (36.6)	137 (63.4)		20 (11.3)	157 (88.7)	
Percentage patient population publicly-insured or uninsured							
<80%	726	99 (29.2)	240 (70.8)	<0.001	25 (6.5)	362 (93.5)	0.04
≥80%	295	94 (50.0)	94 (50.0)		14 (13.1)	93 (86.9)	

© 2020 De Marchis EH et al. *JAMA Network Open*.

eTable 3. Participant Characteristics by Interest in Food Assistance and Food Insecurity Screening Results (N = 1011)

Variable	Total	Screened positive for food insecurity (N=417, 41.3%)		Fisher's exact p value	Screened negative for food insecurity (N=594, 58.7%)		Fisher's exact p value
		Interested in food assistance (N=144, 34.5%) No. (%)	Not interested in food assistance (N=273, 65.5%) No. (%)		Interested in food assistance (N=22, 3.7%) No. (%)	Not interested in food assistance (N=572, 96.3%) No. (%)	
Participant characteristics							
Age (years)							
18-44	541	83 (34.4)	159 (65.7)	0.99	14 (4.7)	285 (95.3)	
45-64	294	45 (34.9)	84 (65.1)		7 (4.2)	158 (95.8)	
≥65	162	14 (35.0)	26 (65.0)		1 (0.8)	121 (99.2)	0.13
Sex							
Female	701	98 (32.9)	200 (61.1)	0.17	15 (3.7)	388 (96.3)	1.00
Male	293	46 (40.4)	68 (59.6)		7 (3.9)	172 (96.1)	
Race/Ethnicity							
Non-Hispanic White	357	34 (36.2)	60 (63.8)	0.17	5 (1.9)	258 (98.1)	0.02
Non-Hispanic Black	210	34 (33.0)	69 (67.0)		8 (7.5)	99 (92.5)	
Hispanic	307	50 (31.3)	110 (68.7)		5 (3.4)	142 (96.6)	
Non-Hispanic Other/Multiple Races	83	17 (51.5)	16 (48.5)		4 (8.0)	46 (92.0)	
Preferred Language							
English	842	114 (36.4)	199 (63.6)	0.19	19 (3.6)	510 (96.4)	0.72
Spanish	169	30 (28.9)	74 (71.1)		3 (4.6)	62 (95.4)	
Education							
<12 years	177	37 (33.0)	75 (67.0)	0.64	1 (1.5)	64 (98.5)	0.50
≥12 years	822	107 (35.7)	193 (64.3)		21 (4.0)	501 (96.0)	

© 2020 De Marchis EH et al. *JAMA Network Open*.

eTable 3. Continued							
Variable	Total	Screened positive for food insecurity (N=417, 41.3%)		Fisher's exact p value	Screened negative for food insecurity (N=594, 58.7%)		Fisher's exact p value
		Interested in food assistance (N=144, 34.5%) No. (%)	Not interested in food assistance (N=273, 65.5%) No. (%)		Interested in food assistance (N=22, 3.7%) No. (%)	Not interested in food assistance (N=572, 96.3%) No. (%)	
Income							
Missing	166	20 (32.3%)	42 (67.7%)	0.13	0 (0.0%)	104 (100.0%)	<0.001
$0-$10,000	222	65 (42.8%)	87 (52.2%)		5 (7.1%)	65 (92.9%)	
$10,001-$25,000	178	32 (29.1%)	78 (70.9%)		8 (10.5%)	68 (89.5%)	
$25,001-$50,000	180	23 (30.3%)	53 (69.7%)		7 (6.7%)	97 (93.3%)	
$50,001-$75,000	80	4 (30.8%)	9 (69.2%)		2 (3.0%)	65 (97.0%)	
≥$75001	177	0 (0.0%)	4 (100.0%)		0 (0.0%)	173 (100.0%)	
Self-Reported Health or Child's Health							
Excellent/Very good/Good	741	87 (32.8)	178 (67.2)	0.27	17 (3.6)	459 (96.4)	0.56
Fair/Poor	235	52 (38.5)	83 (61.5)		5 (5.0)	95 (95.0)	
Participant type							
Adult patient	784	102 (32.6)	211 (67.4)	0.16	12 (2.6)	459 (97.4)	0.007
Adult caregiver of pediatric patient	227	42 (40.4)	62 (59.6)		10 (8.1)	113 (91.9)	
Trust in clinician							
Complete (10)	500	72 (37.7)	119 (62.3)	0.72	9 (2.9)	300 (97.1)	0.22
High (8-9)	283	34 (33.3)	68 (66.7)		7 (3.9)	174 (96.1)	
Medium-Low (1-7)	116	36 (34.0)	70 (66.0)		6 (7.1)	79 (92.9)	

© 2020 De Marchis EH et al. *JAMA Network Open*.

eTable 3. Continued							
Variable	Total	Screened positive for food insecurity (N=417, 41.3%)		Fisher's exact p value	Screened negative for food insecurity (N=594, 58.7%)		Fisher's exact p value
		Interested in food assistance (N=144, 34.5%) No. (%)	Not interested in food assistance (N=273, 65.5%) No. (%)		Interested in food assistance (N=22, 3.7%) No. (%)	Not interested in food assistance (N=572, 96.3%) No. (%)	
Any experience prior discrimination within health care							
Yes	273	49 (33.8)	96 (66.2)	0.83	8 (6.3)	120 (93.7)	0.11
No	722	93 (35.2)	171 (64.8)		14 (3.1)	444 (96.9)	
Ordering of questions about social risk screening and question about interest in assistance							
Answered questions about social risk factors first	505	65 (31.4)	142 (68.6)	0.22	9 (3.0)	289 (97.0)	0.40
Answered question about interest in assistance first	506	79 (37.6)	131 (62.4)		13 (4.4)	283 (95.6)	
Social Risk Screening							
Number of risk factors							
0	358	-	-	<0.001	8 (2.2)	350 (97.8)	0.08
1	250	11 (17.7)	51 (82.3)		11 (5.9)	177 (94.1)	
2	225	51 (28.7)	127 (71.3)		3 (6.4)	44 (93.6)	
3	135	54 (40.3)	80 (59.7)		0 (0.0)	1 (100.0)	
4	40	26 (65.0)	14 (35.0)		-	-	
5	3	2 (66.7)	1 (33.3)		-	-	
Any prior social risk screening exposure in a health care setting in the past 12 months							
Yes	311	67 (40.8)	97 (59.2)	0.03	6 (4.1)	141 (95.9)	0.81
No	682	75 (30.4)	172 (69.6)		16 (3.7)	419 (96.3)	

© 2020 De Marchis EH et al. *JAMA Network Open*.

eTable 3. Continued							
Variable	Total	Screened positive for food insecurity (N=417, 41.3%)		Fisher's exact p value	Screened negative for food insecurity (N=594, 58.7%)		Fisher's exact p value
		Interested in food assistance (N=144, 34.5%) No. (%)	Not interested in food assistance (N=273, 65.5%) No. (%)		Interested in food assistance (N=22, 3.7%) No. (%)	Not interested in food assistance (N=572, 96.3%) No. (%)	
Any prior social risk assistance from health care setting in the past 12 months							
Yes	180	60 (45.1)	73 (54.9)	0.002	5 (10.4)	42 (89.6)	0.03
No	813	81 (29.2)	196 (70.8)		17 (3.2)	519 (96.8)	
Any discomfort with questions in any screening domains							
Yes	68	19 (38.0)	31 (62.0)	0.64	4 (22.2)	14 (77.8)	0.003
No	921	122 (34.3)	234 (65.7)		18 (3.2)	547 (96.8)	
Appropriateness of health care-based social risk screening							
Very/somewhat appropriate	776	114 (34.6)	216 (65.5)	0.78	20 (4.5)	426 (95.5)	0.19
Neither/Very/ somewhat inappropriate	197	26 (36.6)	45 (63.4)		2 (1.6)	124 (98.4)	
Comfort with integrating social risk data in EHR							
Completely/ somewhat comfortable	633	100 (37.9)	164 (62.1)	0.10	16 (4.3)	353 (95.7)	0.66
Neither/completely/ somewhat uncomfortable	247	40 (29.2)	97 (70.8)		4 (3.6)	106 (96.4)	

© 2020 De Marchis EH et al. *JAMA Network Open*.

eTable 3. Continued							
Variable	Total	Screened positive for food insecurity (N=417, 41.3%)		Fisher's exact p value	Screened negative for food insecurity (N=594, 58.7%)		Fisher's exact p value
		Interested in food assistance (N=144, 34.5%) No. (%)	Not interested in food assistance (N=273, 65.5%) No. (%)		Interested in food assistance (N=22, 3.7%) No. (%)	Not interested in food assistance (N=572, 96.3%) No. (%)	
Health care setting characteristics							
Primary Care	622	85 (33.6)	168 (66.4)	0.67	7 (1.9)	362 (98.1)	0.006
Emergency Department	389	59 (36.0)	105 (64.0)		15 (6.7)	210 (93.3)	
Percentage patient population publicly-insured or uninsured							
<80%	720	87 (33.9)	170 (66.1)	0.75	13 (2.8)	450 (97.2)	0.04
≥80%	291	57 (35.6)	103 (64.4)		9 (6.9)	122 (93.1)	

© 2020 De Marchis EH et al. *JAMA Network Open*.

eTable 4. Participant Characteristics by Interest in Utilities Assistance and Utilities Screening Results (N = 1001)

Variable	Total	Screened positive for utilities problem (N=129, 12.9%)		Fisher's exact p value	Screened negative for utilities problem (N=872, 87.1%)		Fisher's exact p value
		Interested in utilities assistance (N=45, 34.9%) No. (%)	Not interested in utilities assistance (N=84, 65.1%) No. (%)		Interested in utilities assistance (N=66, 7.6%) No. (%)	Not interested in utilities assistance (N=806, 92.4%) No. (%)	
Participant characteristics							
Age (years)							
18-44	536	28 (35.4)	51 (64.6)	0.58	34 (7.4)	423 (92.6)	0.72
45-64	292	16 (38.1)	26 (61.9)		22 (8.8)	228 (91.2)	
≥65	160	1 (14.3)	6 (85.7)		10 (6.5)	143(93.5)	
Sex							
Female	695	41 (41.4)	58 (58.6)	0.01	44 (7.4)	552 (92.6)	0.68
Male	289	4 (14.8)	23 (85.2)		22 (8.4)	240 (91.6)	
Race/Ethnicity							
Non-Hispanic White	352	8 (22.2)	28 (77.8)	0.03	21 (6.7)	295 (93.3)	0.59
Non-Hispanic Black	209	22 (53.7)	19 (46.3)		17 (10.1)	151 (89.9)	
Hispanic	301	11 (30.6)	25 (69.4)		20 (7.6)	245 (92.4)	
Non-Hispanic Other/Multiple Races	84	2 (28.6)	5 (71.4)		6 (7.8)	71 (92.2)	
Preferred Language							
English	838	40 (36.7)	69 (63.3)	0.45	53 (7.3)	676 (92.7)	0.49
Spanish	163	5 (25.0)	15 (75.0)		13 (9.1)	130 (90.9)	
Education							
<12 years	172	10 (28.5)	16 (61.5)	0.82	10 (6.9)	136 (93.1)	0.86
≥12 years	817	35 (34.3)	67 (65.7)		55 (7.7)	660 (92.3)	

© 2020 De Marchis EH et al. *JAMA Network Open*.

eTable 4. Continued							
Variable	Total	Screened positive for utilities problem (N=129, 12.9%)		Fisher's exact p value	Screened negative for utilities problem (N=872, 87.1%)		Fisher's exact p value
		Interested in utilities assistance (N=45, 34.9%) No. (%)	Not interested in utilities assistance (N=84, 65.1%) No. (%)		Interested in utilities assistance (N=66, 7.6%) No. (%)	Not interested in utilities assistance (N=806, 92.4%) No. (%)	
Income							
Missing	158	3 (27.3)	8 (72.7)	0.16	5 (3.4)	142 (96.6)	<0.001
$0-$10,000	222	21 (44.7%)	26 (55.3%)		24 (13.7%)	151 (86.3%)	
$10,001-$25,000	185	10 (37.0%)	17 (63.0%)		19 (12.0%)	139 (88.0%)	
$25,001-$50,000	181	10 (34.5%)	19 (65.5%)		14 (9.2%)	138 (90.8%)	
$50,001-$75,000	80	1 (14.3%)	6 (85.7%)		3 (4.1%)	70 (95.9%)	
≥$75001	175	0 (0.0%)	8 (100.0%)		1 (0.6%)	166 (99.4%)	
Self-Reported Health or Child's Health							
Excellent/Very good/Good	736	26 (29.9)	61 (70.1)	0.05	42 (6.5)	607 (93.5)	0.03
Fair/Poor	202	19 (48.7)	0 (51.3)		22 (11.5)	170 (88.5)	
Participant type							
Adult patient	776	25 (28.1)	64 (71.9)	0.03	50 (7.3)	637 (92.7)	0.53
Adult caregiver of pediatric patient	225	20 (50.0)	20 (50.0)		16 (8.7)	169 (91.4)	
Trust in clinician							
Complete (10)	498	25 (38.5)	40 (61.5)	0.78	27 (62)	406 (93.8)	0.18
High (8-9)	282	11 (33.3)	22 (66.7)		20 (8.0)	229 (92.0)	
Medium-Low (1-7)	188	9 (31.0)	20 (69.0)		17 (10.7)	142 (89.3)	

© 2020 De Marchis EH et al. *JAMA Network Open*.

eTable 4. Continued							
Variable	Total	Screened positive for utilities problem (N=129, 12.9%)		Fisher's exact p value	Screened negative for utilities problem (N=872, 87.1%)		Fisher's exact p value
		Interested in utilities assistance (N=45, 34.9%) No. (%)	Not interested in utilities assistance (N=84, 65.1%) No. (%)		Interested in utilities assistance (N=66, 7.6%) No. (%)	Not interested in utilities assistance (N=806, 92.4%) No. (%)	
Any experience prior discrimination within health care							
Yes	269	14 (28.6)	35 (71.4)	0.19	16 (7.3)	204 (92.7)	1.00
No	717	31 (40.8)	45 (59.2)		49 (7.6)	592 (92.4)	
Ordering of questions about social risk screening and question about interest in assistance							
Answered questions about social risk factors first	505	22 (36.7)	38 (63.3)	0.72	24 (5.4)	421 (94.6)	0.02
Answered question about interest in assistance first	496	23 (33.3)	46 (66.7)		42 (9.8)	385 (90.2)	
Social Risk Screening							
Number of risk factors							
0	355	-	-	0.34	6 (1.7)	349 (98.3)	<0.001
1	238	4 (22.2)	1 (77.8)		15 (6.4)	218 (93.6)	
2	222	9 (29.0)	22 (71.0)		29 (15.2)	162 (84.8)	
3	130	15 (34.1)	29 (65.9)		15 (17.4)	71 (82.6)	
4	40	16 (48.5)	17 (51.5)		1 (14.3)	6 (85.7)	
5	3	1 (33.3)	2 (66.7)		-	-	
Any prior social risk screening exposure in a health care setting in the past 12 months							
Yes	307	16 (30.8)	36 (69.2)	0.45	30 (11.8)	225 (88.2)	0.003
No	679	29 (38.2)	47 (61.8)		34 (5.6)	569 (94.4)	

© 2020 De Marchis EH et al. *JAMA Network Open*.

eTable 4. Continued							
Variable	Total	Screened positive for utilities problem (N=129, 12.9%)		Fisher's exact p value	Screened negative for utilities problem (N=872, 87.1%)		Fisher's exact p value
		Interested in utilities assistance (N=45, 34.9%) No. (%)	Not interested in utilities assistance (N=84, 65.1%) No. (%)		Interested in utilities assistance (N=66, 7.6%) No. (%)	Not interested in utilities assistance (N=806, 92.4%) No. (%)	
Any prior social risk assistance from health care setting in the past 12 months							
Yes	180	16 (43.2)	21 (56.8)	0.23	22 (15.4)	121 (84.6)	<0.001
No	807	29 (31.9)	62 (68.1)		44 (6.2)	672 (93.8)	
Any discomfort with questions in any screening domains							
Yes	65	3 (20.0)	12 (80.0)	0.26	8 (16.0)	42 (84.0)	0.05
No	916	42 (37.2)	71 (62.8)		57 (7.1)	746 (92.9)	
Appropriateness of health care-based social risk screening							
Very/somewhat appropriate	773	37 (35.6)	67 (64.4)	0.81	51 (7.6)	618 (92.4)	0.74
Neither/Very/somewhat inappropriate	193	8 (38.1)	13 (61.9)		11 (6.4)	161 (93.6)	
Comfort with integrating social risk data in EHR							
Completely/somewhat comfortable	631	28 (38.4)	45 (61.6)	0.57	46 (8.2)	512 (91.8)	0.14
Neither/completely/somewhat uncomfortable	206	17 (32.7)	35 (67.3)		12 (7.8)	142 (92.2)	

© 2020 De Marchis EH et al. *JAMA Network Open*.

eTable 4. Continued							
Variable	Total	Screened positive for utilities problem (N=129, 12.9%)		Fisher's exact p value	Screened negative for utilities problem (N=872, 87.1%)		Fisher's exact p value
		Interested in utilities assistance (N=45, 34.9%) No. (%)	Not interested in utilities assistance (N=84, 65.1%) No. (%)		Interested in utilities assistance (N=66, 7.6%) No. (%)	Not interested in utilities assistance (N=806, 92.4%) No. (%)	
Health care setting characteristics							
Primary Care	613	23 (29.9)	54 (70.1)	0.19	40 (7.5)	496 (92.5)	0.90
Emergency Department	388	22 (42.3)	30 (57.7)		26 (7.7)	310 (92.3)	
Percentage patient population publicly-insured or uninsured							
<80%	713	26 (29.9)	61 (70.1)	0.12	49 (7.8)	577 (92.2)	0.78
≥80%	288	19 (45.2)	23 (54.8)		17 (6.9)	229 (93.1)	

© 2020 De Marchis EH et al. *JAMA Network Open*.

eTable 5. Participant Characteristics by Interest in Transportation Assistance and Transportation Problems Screening Results (N =998)

Variable	Total	Screened positive for transportation problems (N=200, 20.0%)		Fisher's exact p value	Screened negative for transportation problems (N=798, 80.0%)		Fisher's exact p value
		Interested in transportation assistance (N=79, 39.5%) No. (%)	Not interested in transportation assistance (N=121, 60.5%) No. (%)		Interested in transportation assistance (N=39, 4.9%) No. (%)	Not interested in transportation assistance (N=759, 95.1%) No. (%)	
Participant characteristics							
Age (years)							
18-44	534	45 (37.5)	75 (62.5)	0.68	16 (3.9)	398 (96.1)	0.26
45-64	291	24 (44.4)	30 (55.6)		16 (6.8)	221 (93.2)	
≥65	160	10 (41.7)	14 (58.3)		7 (5.2)	129 (94.9)	
Sex							
Female	695	57 (38.5)	91 (61.5)	0.74	17 (3.1)	530 (96.9)	0.003
Male	286	21 (42.0)	29 (58.0)		20 (8.5)	216 (91.5)	
Race/Ethnicity							
Non-Hispanic White	331	15 (37.5)	2 (62.5)	0.97	4 (1.3)	310 (98.7)	0.001
Non-Hispanic Black	208	19 (38.8)	30 (61.2)		11 (6.9)	148 (93.1)	
Hispanic	300	32 (40.5)	47 (59.5)		15 (6.8)	206 (93.2)	
Non-Hispanic Other/Multiple Races	82	8 (44.4)	10 (55.6)		5 (7.8)	59 (92.2)	
Preferred Language							
English	271	59 (38.6)	94 (61.4)	0.73	31 (4.6)	650 (95.4)	0.35
Spanish	164	20 (42.6)	27 (57.4)		8 (6.8)	109 (93.2)	
Education							
<12 years	171	18 (34.0)	35 (66.0)	0.41	13 (11.0)	105 (89.0)	0.004
≥12 years	761	6 (41.5)	86 (58.5)		26 (3.9)	643 (96.1)	

© 2020 De Marchis EH et al. *JAMA Network Open*.

eTable 5. Continued							
Variable	Total	Screened positive for transportation problems (N=200, 20.0%)		Fisher's exact p value	Screened negative for transportation problems (N=798, 80.0%)		Fisher's exact p value
		Interested in transportation assistance (N=79, 39.5%) No. (%)	Not interested in transportation assistance (N=121, 60.5%) No. (%)		Interested in transportation assistance (N=39, 4.9%) No. (%)	Not interested in transportation assistance (N=759, 95.1%) No. (%)	
Income							
Missing	160	12 (36.4)	21 (63.6)	0.97	7 (5.5)	120 (94.5)	0.001
$0-$10,000	218	35 (41.2%)	50 (58.8%)		13 (9.8%)	120 (90.2%)	
$10,001-$25,000	186	14 (36.8%)	24 (63.2%)		12 (8.1%)	136 (91.9%)	
$25,001-$50,000	179	14 (42.4%)	19 (57.6%)		4 (2.7%)	142 (97.3%)	
$50,001-$75,000	79	2 (50.0%)	2 (50.0%)		2 (2.7%)	73 (97.3%)	
≥$75001	176	2 (28.6%)	5 (71.4%)		1 (0.6%)	168 (99.4%)	
Self-Reported Health or Child's Health							
Excellent/Very good/Good	732	45 (36.6)	78 (63.4)	0.29	23 (3.8)	586 (96.2)	0.02
Fair/Poor	234	32 (45.1)	39 (54.9)		14 (9.6)	149 (91.4)	
Participant type							
Adult patient	775	60 (39.2)	93 (60.8)	1.00	33 (5.3)	589 (94.7)	0.43
Adult caregiver of pediatric patient	223	19 (40.4)	28 (59.6)		6 (3.4)	170 (96.6)	
Trust in clinician							
Complete (10)	495	32 (39.0)	50 (61.0)	0.64	12 (2.9)	401 (97.1)	0.81
High (8-9)	280	22 (45.8)	26 (54.2)		11 (4.7)	221 (95.3)	
Medium-Low (1-7)	154	24 (37.5)	40 (62.5)		14 (11.1)	112 (88.9)	

© 2020 De Marchis EH et al. *JAMA Network Open*.

eTable 5. Continued							
Variable	Total	Screened positive for transportation problems (N=200, 20.0%)		Fisher's exact p value	Screened negative for transportation problems (N=798, 80.0%)		Fisher's exact p value
		Interested in transportation assistance (N=79, 39.5%) No. (%)	Not interested in transportation assistance (N=121, 60.5%) No. (%)		Interested in transportation assistance (N=39, 4.9%) No. (%)	Not interested in transportation assistance (N=759, 95.1%) No. (%)	
Any experience prior discrimination within health care							
Yes	479	27 (35.1)	50 (64.9)	0.30	14 (7.2)	180 (92.8)	0.08
No	711	51 (42.9)	68 (57.1)		23 (3.9)	569 (96.1)	
Ordering of questions about social risk screening and question about interest in assistance							
Answered questions about social risk factors first	505	38 (36.9)	65 (63.1)	0.47	18 (4.5)	384 (95.5)	0.63
Answered question about interest in assistance first	493	41 (42.3)	56 (57.7)		21 (5.3)	375 (94.7)	
Social Risk Screening							
Number of risk factors							
0	352	-	-	0.97	11 (3.1)	341 (96.9)	0.04
1	247	8 (44.4)	10 (55.6)		12 (5.2)	217 (94.8)	
2	224	19 (37.3)	32 (62.7)		10 (5.8)	163 (94.2)	
3	132	35 (38.9)	55 (61.1)		6 (14.3)	36 (85.7)	
4	58	16 (42.1)	22 (57.9)		0 (0.0)	2 (100.0)	
5	3	1 (33.3)	2 (66.7)		-	-	
Any prior social risk screening exposure in a health care setting in the past 12 months							
Yes	306	35 (39.8)	53 (60.2)	0.88	9 (4.1)	209 (95.9)	0.71
No	677	41 (38.3)	66 (61.7)		28 (4.9)	542 (95.1)	

© 2020 De Marchis EH et al. *JAMA Network Open*.

eTable 5. Continued							
Variable	Total	Screened positive for transportation problems (N=200, 20.0%)		Fisher's exact p value	Screened negative for transportation problems (N=798, 80.0%)		Fisher's exact p value
		Interested in transportation assistance (N=79, 39.5%) No. (%)	Not interested in transportation assistance (N=121, 60.5%) No. (%)		Interested in transportation assistance (N=39, 4.9%) No. (%)	Not interested in transportation assistance (N=759, 95.1%) No. (%)	
Any prior social risk assistance from health care setting in the past 12 months							
Yes	177	29 (39.7)	44 (60.3)	0.65	13 (12.5)	91 (87.5)	0.001
No	806	44 (36.4)	77 (63.6)		25 (3.7)	660 (96.3)	
Any discomfort with questions in any screening domains							
Yes	68	10 (30.3)	23 (69.7)	0.33	3 (8.6)	32 (91.4)	0.23
No	845	64 (40.0)	96 (60.0)		34 (4.6)	714 (95.4)	
Appropriateness of health care-based social risk screening							
Very/somewhat appropriate	337	59 (38.6)	94 (61.4)	0.86	33 (3.2)	151 (96.8)	0.31
Neither/Very/ somewhat inappropriate	626	16 (40.0)	24 (60.0)		5 (3.1)	581 (94.6)	
Comfort with integrating social risk data in EHR							
Completely/ somewhat comfortable	622	58 (43.0)	77 (57.0)	0.08	29 (6.0)	458 (94.0)	0.12
Neither/completely/ somewhat uncomfortable	317	17 (29.3)	41 (70.7)		9 (3.2)	274 (96.8)	

© 2020 De Marchis EH et al. *JAMA Network Open*.

eTable 5. Continued							
Variable	Total	Screened positive for transportation problems (N=200, 20.0%)		Fisher's exact p value	Screened negative for transportation problems (N=798, 80.0%)		Fisher's exact p value
		Interested in transportation assistance (N=79, 39.5%) No. (%)	Not interested in transportation assistance (N=121, 60.5%) No. (%)		Interested in transportation assistance (N=39, 4.9%) No. (%)	Not interested in transportation assistance (N=759, 95.1%) No. (%)	
Health care setting characteristics							
Primary Care	610	48 (40.3)	71 (59.7)	0.88	20 (4.1)	471 (95.9)	0.19
Emergency Department	388	31 (38.3)	50 (61.7)		19 (6.2)	288 (93.8)	
Percentage patient population publicly-insured or uninsured							
<80%	715	50 (42.0)	69 (58.0)	0.46	21 (3.5)	575 (96.5)	0.004
≥80%	283	29 (35.8)	52 (64.2)		18 (8.9)	184 (91.1)	

© 2020 De Marchis EH et al. *JAMA Network Open*.

eTable 6. Participant Characteristics by Interest in Safety Assistance and Interpersonal Violence Screening Results (N = 984)

Variable	Total	Screened positive for interpersonal violence (N=18, 1.8%)		Fisher's exact p value	Screened negative for interpersonal violence (N=966, 98.2%)		Fisher's exact p value
		Interested in safety assistance (N=2, 11.1%) No. (%)	Not interested in safety assistance (N=16, 88.9%) No. (%)		Interested in safety assistance (N=16, 1.7%) No. (%)	Not interested in safety assistance (N=950, 98.3%) No. (%)	
Participant characteristics							
Age (years)							
18-44	531	0 (0.0)	10 (100.0)	0.25	10 (1.9)	511 (98.1)	0.94
45-64	284	2 (28.6)	5 (71.4)		4 (1.4)	273 (98.6)	
≥65	156	0 (0.0)	1 (100.0)		2 (1.3)	153 (98.7)	
Sex							
Female	687	0 (0.0)	11 (100.0)	0.14	11 (1.6)	665 (98.4)	0.79
Male	280	2 (28.6)	5 (71.4)		5 (1.8)	268 (98.2)	
Race/Ethnicity							
Non-Hispanic White	346	1 (20.0)	4 (80.0)	0.65	0 (0.0)	341 (100.0)	0.004
Non-Hispanic Black	208	0 (0.0)	3 (100.0)		6 (2.9)	199 (97.1)	
Hispanic	296	1 (25.0)	3 (75.0)		6 (2.1)	286 (97.9)	
Non-Hispanic Other/Multiple Races	82	0 (0.0)	6 (100.0)		2 (2.6)	74 (97.4)	
Preferred Language							
English	826	1 (6.3)	15 (93.7)	0.22	14 (1.7)	796 (98.3)	1.00
Spanish	158	1 (50.0)	1 (50.0)		2 (1.3)	154 (98.7)	
Education							
<12 years	167	0 (0.0)	4 (100.0)	1.00	2 (1.2)	161 (98.8)	1.00
≥12 years	805	2 (14.3)	12 (85.7)		14 (1.8)	777 (98.2)	

© 2020 De Marchis EH et al. *JAMA Network Open*.

eTable 6. Continued							
Variable	Total	Screened positive for interpersonal violence (N=18, 1.8%)		Fisher's exact p value	Screened negative for interpersonal violence (N=966, 98.2%)		Fisher's exact p value
		Interested in safety assistance (N=2, 11.1%) No. (%)	Not interested in safety assistance (N=16, 88.9%) No. (%)		Interested in safety assistance (N=16, 1.7%) No. (%)	Not interested in safety assistance (N=950, 98.3%) No. (%)	
Income							
Missing	159	0 (0.0)	3 (100.0)	0.31	1 (0.6)	155 (99.4)	0.005
$0-$10,000	216	1 (10.0%)	9 (90.0%)		10 (4.9%)	196 (95.1%)	
$10,001-$25,000	179	0 (0.0%)	3 (100.0%)		3 (1.7%)	173 (98.3%)	
$25,001-$50,000	178	0 (0.0%)	1 (100.0%)		1 (0.6%)	176 (99.4)	
$50,001-$75,000	79	1 (100.0%)	0 (0.0%)		1 (1.3%)	77 (98.7%)	
≥$75001	173	-	-		0 (0.0%)	173 (100.0%)	
Self-Reported Health or Child's Health							
Excellent/Very good/Good	226	0 (0.0)	7 (100.0)	0.49	6 (2.7)	213 (97.3)	0.23
Fair/Poor	726	2 (20.0)	8 (80.0)		10 (1.4)	706 (98.6)	
Participant type							
Adult patient	759	1 (7.1)	13 (92.9)	0.41	11 (1.5)	734 (98.5)	0.38
Adult caregiver of pediatric patient	225	1 (25.0)	3 (75.0)		5 (2.3)	216 (97.7)	
Trust in clinician							
Complete (10)	485	2 (18.2)	9 (81.8)	0.57	9 (1.9)	465 (98.1)	0.59
High (8-9)	280	0 (0.0)	1 (100.0)		3 (1.1)	276 (98.9)	
Medium-Low (1-7)	184	0 (0.0)	6 (100.0)		4 (2.3)	174 (97.7)	

© 2020 De Marchis EH et al. *JAMA Network Open*.

eTable 6. Continued							
Variable	Total	Screened positive for interpersonal violence		Fisher's exact p value	Screened negative for interpersonal violence		Fisher's exact p value
		(N=18, 1.8%)			(N=966, 98.2%)		
		Interested in safety assistance	Not interested in safety assistance		Interested in safety assistance	Not interested in safety assistance	
		(N=2, 11.1%) No. (%)	(N=16, 88.9%) No. (%)		(N=16, 1.7%) No. (%)	(N=950, 98.3%) No. (%)	
Any experience prior discrimination within health care							
Yes	264	2 (25.0)	6 (75.0)	1.00	4 (1.6)	252 (98.4)	1.00
No	704	0 (0.0)	10 (100.0)		12 (1.7)	682 (98.3)	
Ordering of questions about social risk screening and question about interest in assistance							
Answered questions about social risk factors first	494	2 (16.7)	10 (83.3)	0.53	5 (1.0)	477 (99.0)	0.21
Answered question about interest in assistance first	490	0 (0.0)	6 (100.0)		11 (2.3)	473 (97.7)	
Social Risk Screening							
Number of risk factors							
0	349	-	-	0.71	2 (0.6)	347 (99.4)	0.01
1	247	0 (0.0)	1 (100.0)		4 (1.6)	243 (98.4)	
2	219	-	-		4 (1.8)	215 (98.2)	
3	128	0 (0.0)	5 (100.0)		3 (2.4)	120 (97.6)	
4	37	2 (22.2)	7 (77.8)		3 (10.7)	25 (89.3)	
5	3	0 (0.0)	3 (100.0)		-	-	
Any prior social risk screening exposure in a health care setting in the past 12 months							
Yes	301	2 (18.2)	9 (81.8)	0.50	5 (1.7)	285 (98.3)	1.00
No	667	0 (0.0)	7 (100.0)		11 (1.7)	649 (98.3)	

© 2020 De Marchis EH et al. *JAMA Network Open*.

eTable 6. Continued							
Variable	Total	Screened positive for interpersonal violence (N=18, 1.8%)		Fisher's exact p value	Screened negative for interpersonal violence (N=966, 98.2%)		Fisher's exact p value
		Interested in safety assistance (N=2, 11.1%) No. (%)	Not interested in safety assistance (N=16, 88.9%) No. (%)		Interested in safety assistance (N=16, 1.7%) No. (%)	Not interested in safety assistance (N=950, 98.3%) No. (%)	
Any prior social risk assistance from health care setting in the past 12 months							
Yes	169	1 (12.5)	7 (87.5)	1.00	4 (2.5)	157 (97.5)	0.33
No	800	1 (10.0)	9 (90.0)		12 (1.5)	778 (98.5)	
Any discomfort with questions in any screening domains							
Yes	66	1 (20.0)	4 (80.0)	0.49	1 (1.6)	60 (98.4)	1.00
No	901	1 (7.7)	12 (92.3)		15 (1.7)	873 (98.3)	
Appropriateness of health care-based social risk screening							
Very/somewhat appropriate	754	2 (13.3)	13 (86.7)	1.00	14 (1.9)	725 (98.1)	0.33
Neither/Very/ somewhat inappropriate	195	0 (0.0)	3 (100.0)		1 (0.5)	191 (99.5)	
Comfort with integrating social risk data in EHR							
Completely/ somewhat comfortable	615	2 (15.4)	11 (84.6)	1.00	13 (2.2)	589 (97.8)	0.09
Neither/completely/ somewhat uncomfortable	188	0 (0.0)	5 (100.0)		0 (0.0)	183 (100.0)	

© 2020 De Marchis EH et al. *JAMA Network Open*.

eTable 6. Continued							
Variable	Total	Screened positive for interpersonal violence		Fisher's exact p value	Screened negative for interpersonal violence		Fisher's exact p value
		(N=18, 1.8%)			(N=966, 98.2%)		
		Interested in safety assistance	Not interested in safety assistance		Interested in safety assistance	Not interested in safety assistance	
		(N=2, 11.1%) No. (%)	(N=16, 88.9%) No. (%)		(N=16, 1.7%) No. (%)	(N=950, 98.3%) No. (%)	
Health care setting characteristics							
Primary Care	600	1 (8.3)	11 (91.7)	1.00	9 (1.5)	579 (98.5)	0.79
Emergency Department	384	1 (16.7)	5 (83.3)		7 (1.9)	371 (98.1)	
Percentage patient population publicly-insured or uninsured							
<80%	701	2 (22.2)	7 (77.8)	1.00	9 (1.3)	683 (98.7)	0.17
≥80%	283	0 (0.0)	9 (100.0)		7 (2.6)	267 (97.5)	

© 2020 De Marchis EH et al. *JAMA Network Open*.

eTable 7. Differences in Participant Characteristics, Stratified by Social Risk Screening Results, Between Respondents Included in Multivariable Model (N = 851) and Respondents Excluded Owing to Missingness (N = 170)

Variable (N[a])	Screened Positive for 1+ Risk (N=662)			Screened Negative for All Risks (N=359)		
	No missing data in multivariable model (N=550, 83%) No. (%)	Missing data in multivariable model (N=112, 17%) No. (%)	Fisher's exact p value	No missing data in multivariable model (N=301, 84%) No. (%)	Missing data in multivariable model (N=58, 16%) No. (%)	Fisher's exact p value
Participant characteristics						
Age (years) (1007)						
18-44	332 (60.4%)	49 (48.0%)	0.04	145 (48.2%)	18 (33.3%)	0.01
45-64	159 (28.9%)	35 (34.3%)		91 (30.2%)	14 (25.9%)	
≥65	59 (10.7%)	18 (17.7%)		65 (21.6%)	22 (40.7%)	
Sex (1004)						
Female	399 (73.2%)	67 (63.8%)	0.06	207 (69.2%)	36 (65.5%)	0.64
Male	146 (26.8%)	38 (36.2%)		92 (30.8%)	19 (34.5%)	
Race/Ethnicity (965)						
Non-Hispanic White	175 (31.8%)	10 (14.3)	<0.001	157 (52.2%)	17 (38.6%)	0.27
Non-Hispanic Black	140 (25.5%)	10 (14.3)		53 (17.6%)	8 (18.2%)	
Hispanic	187 (34.0%)	46 (65.7%)		65 (21.6%)	13 (29.6%)	
Non-Hispanic Other/Multiple Races	48 (8.7%)	4 (5.7)		26 (8.6%)	6 (13.6%)	
Preferred Language (1021)						
English	451 (82.0%)	70 (62.5%)	<0.001	278 (92.4%)	49 (84.5%)	0.07
Spanish	99 (18.0%)	42 (37.5%)		23 (7.6%)	9 (15.5%)	
Education (1009)						
<12 years	100 (18.2%)	46 (44.2%)	<0.001	23 (7.6%)	12 (22.2%)	0.002
≥12 years	450 (81.8%)	58 (55.8%)		278 (92.4%)	42 (77.8%)	

© 2020 De Marchis EH et al. *JAMA Network Open*.

eTable 7. Continued						
Variable (N[a])	Screened Positive for 1+ Risk (N=662)		Fisher's exact p value	Screened Negative for All Risks (N=359)		Fisher's exact p value
	No missing data in multivariable model	Missing data in multivariable model		No missing data in multivariable model	Missing data in multivariable model	
	(N=550, 83%) No. (%)	(N=112, 17%) No. (%)		(N=301, 84%) No. (%)	(N=58, 16%) No. (%)	
Income (853)						
$0-10,000	164 (34.0%)	31 (41.3%)	0.01	26 (10.0%)	4 (11.1%)	0.85
$10,001-25,000	118 (24.4%)	28 (37.3%)		38 (14.7%)	5 (13.9%)	
$25,001-50,000	116 (24.0%)	10 (13.3%)		47 (18.2%)	9 (25.0%)	
$50,001-75,000	34 (7.0%)	1 (1.3%)		41 (15.8%)	4 (11.1%)	
>$75,001	51 (10.6%)	5 (6.7%)		104 (41.3%)	14 (38.9%)	
Self-Reported Health or Child's Health (986)						
Excellent/Very good/Good	399 (72.6%)	55 (64.0%)	0.12	257 (85.4%)	37 (75.5%)	0.09
Fair/Poor	151 (27.4%)	31 (36.0%)		44 (14.6%)	12 (24.5%)	
Participant type (1021)						
Adult patient	406 (73.8%)	92 (82.1%)	0.07	240 (79.7%)	55 (94.8%)	0.004
Adult caregiver of pediatric patient	144 (26.2%)	20 (17.9%)		61 (20.3%)	3 (5.2%)	
Trust in clinician (981)						
Complete (10)	269 (48.9%)	44 (51.8%)	0.81	164 (54.5%)	27 (60.0%)	0.16
High (8-9)	154 (28.0%)	24 (28.2%)		98 (32.6%)	9 (20.0%)	
Medium-Low (1-7)	127 (23.1%)	17 (20.0%)		39 (13.0%)	9 (20.0%)	

© 2020 De Marchis EH et al. *JAMA Network Open*.

eTable 7. Continued						
Variable (N[a])	Screened Positive for 1+ Risk (N=662)		Fisher's exact p value	Screened Negative for All Risks (N=359)		Fisher's exact p value
	No missing data in multivariable model	Missing data in multivariable model		No missing data in multivariable model	Missing data in multivariable model	
	(N=550, 83%) No. (%)	(N=112, 17%) No. (%)		(N=301, 84%) No. (%)	(N=58, 16%) No. (%)	
Any experience prior discrimination within health care (1005)						
Yes	175 (31.8%)	36 (35.6%)	0.49	50 (16.6%)	13 (24.5%)	0.18
No	375 (68.2%)	65 (64.4%)		251 (83.4%)	40 (75.5%)	
Ordering of questions about social risk screening and question about interest in assistance (1021)						
Answered questions about social risk factors first	272 (49.5%)	58 (51.8%)	0.68	154 (51.2%)	29 (50.0%)	0.89
Answered question about interest in assistance first	278 (50.5%)	54 (48.2%)		147 (48.8%)	29 (50.0%)	
Social Risk Screening						
Overall social risk (1021)						
No risk factors	-	-	0.99	301 (100.0%)	58 (100.0%)	N/A
1	214 (38.9%)	43 (38.4%)				
2	187 (34.0%)	40 (35.7%)				
3	113 (20.6%)	22 (19.6%)				
4 to 5	36 (6.6%)	7 (6.25%)		-	-	
Any prior social risk screening exposure in a health care setting in the past 12 months (1002)						
Yes	204 (37.1%)	39 (38.2%)	0.82	59 (19.6%)	11 (22.5%)	0.70
No	346 (62.9%)	63 (61.8%)		242 (80.4%)	38 (77.5%)	

© 2020 De Marchis EH et al. *JAMA Network Open*.

eTable 7. Continued						
Variable (N[a])	Screened Positive for 1+ Risk (N=662)		Fisher's exact p value	Screened Negative for All Risks (N=359)		Fisher's exact p value
	No missing data in multivariable model	Missing data in multivariable model		No missing data in multivariable model	Missing data in multivariable model	
	(N=550, 83%) No. (%)	(N=112, 17%) No. (%)		(N=301, 84%) No. (%)	(N=58, 16%) No. (%)	
Any prior social risk assistance from health care setting in the past 12 months (1002)						
Yes	136 (24.7%)	27 (27.0%)	0.62	15 (5.0%)	4 (7.8%)	0.50
No	414 (75.3%)	72 (73.0%)		286 (95.0%)	47 (92.2%)	
Any discomfort with questions in any screening domains (998)						
Yes	46 (8.4%)	16 (16.3%)	0.02	4 (1.3%)	3 (6.1%)	0.06
No	504 (91.6%)	82 (83.7%)		297 (98.7%)	46 (93.9%)	
Interest in any form of assistance (1021)						
Yes	285 (51.8%)	68 (60.7%)	0.10	26 (8.6%)	5 (8.6%)	1.00
No	265 (48.2%)	44 (39.3%)		275 (91.4%)	53 (91.4%)	
Appropriateness of health care-based social risk screening (982)						
Very/somewhat appropriate	444 (80.7%)	71 (81.6%)	1.00	237 (78.7%)	30 (68.2%)	0.13
Neither/very/ somewhat inappropriate	106 (19.3%)	16 (18.4%)		64 (21.3%)	14 (31.8%)	
Comfort with integrating social risk data in EHR (982)						
Completely/somewhat comfortable	344 (62.6%)	59 (67.8%)	0.40	207 (68.8%)	22 (50.0%)	0.02
Neither/completely/ somewhat uncomfortable	206 (37.4%)	28 (32.2%)		94 (31.2%)	22 (50.0%)	

© 2020 De Marchis EH et al. *JAMA Network Open*.

eTable 7. Continued						
Variable (N[a])	Screened Positive for 1+ Risk (N=662)		Fisher's exact p value	Screened Negative for All Risks (N=359)		Fisher's exact p value
	No missing data in multivariable model	Missing data in multivariable model		No missing data in multivariable model	Missing data in multivariable model	
	(N=550, 83%) No. (%)	(N=112, 17%) No. (%)		(N=301, 84%) No. (%)	(N=58, 16%) No. (%)	
Health care setting characteristics (1021)						
Primary Care	322 (58.5%)	76 (67.9%)	0.07	179 (59.5%)	51 (87.9%)	<0.001
Emergency Department	228 (41.5%)	36 (32.1%)		122 (40.5%)	7 (12.1%)	
Percentage patient population publicly-insured or uninsured (1021)						
<80%	370 (67.3%)	65 (58.0%)	0.06	244 (81.1%)	47 (81.0%)	1.00
≥80%	180 (32.7%)	47 (42.0%)		57 (18.9%)	11 (19.0%)	

[a]Number of participants with complete responses for each variable

© 2020 De Marchis EH et al. *JAMA Network Open*.

eTable 8. Pooled Analyses: Unadjusted and Adjusted Associations Between Interest in Assistance Among Participants With 1 or More Social Risk Factor (N = 851)

Variable	Unadjusted OR (95% CI), p value (N=851)	Adjusted OR (95% CI), p value (N=851)
Participant characteristics		
Age (years)		
18-44	Ref	Ref
45-64	0.78 (0.50, 1.21), 0.26	1.21 (0.94, 1.54), 0.13
≥65	0.47 (0.29, 0.75), 0.002	1.40 (0.92, 2.12), 0.12
Race/Ethnicity		
Non-Hispanic White	Ref	Ref
Non-Hispanic Black	3.96 (2.20, 7.15), <0.001	1.83 (1.32, 2.55), <0.001
Hispanic	2.71 (1.86, 3.94), <0.001	0.82 (0.35, 1.93), 0.65
Non-Hispanic Other/Multiple Races	2.46 (1.33, 4.56), 0.004	1.49 (0.93, 2.39), 0.10
Preferred Language		
English	Ref	Ref
Spanish	2.15 (1.35, 3.43), 0.001	2.13 (1.06, 4.25), 0.03
Education		
<12 years	2.28 (1.50, 3.47), <0.001	1.15 (0.70, 1.97), 0.59
≥12 years	Ref	Ref
Income		
Missing	10.2 (4.30, 24.2), <0.001	3.75 (1.11, 12.65), 0.03
\$0-\$10,000	44.5 (27.5, 72.1), <0.001	7.72 (3.82, 15.60), <0.001
\$10,001-\$25,000	17.1 (9.70, 30.2), <0.001	4.30 (2.05, 9.01), <0.001
\$25,001-\$50,000	13.2 (7.82, 22.4), <0.001	4.68 (2.61, 8.38), <0.001
\$50,001-\$75,000	3.32 (1.38, 7.96), 0.007	2.09 (0.78, 5.63), 0.14
≥\$75001	Ref	Ref
Self-Reported Health or Child's Health		
Excellent/Very good/Good	Ref	Ref
Fair/Poor	2.22 (1.40, 3.52), 0.001	1.64 (1.04, 2.57), 0.03
Participant type		
Adult patient	Ref	Ref
Adult caregiver of pediatric patient	1.96 (0.92, 4.18), 0.08	1.29 (0.63, 2.66), 0.49
Trust in clinician		
Complete (10)	0.57 (0.36, 0.90), 0.02	1.06 (0.62, 1.80), 0.84
High (8-9)	0.55 (0.37, 0.82), 0.003	1.17 (0.70, 1.97), 0.55
Medium-Low (1-7)	Ref	Ref

© 2020 De Marchis EH et al. *JAMA Network Open*.

eTable 8. Continued		
	Unadjusted OR (95% CI), p value	Adjusted OR (95% CI), p value
Variable	(N=851)	(N=851)
Any experience prior discrimination within health care		
Yes	1.42 (1.04, 1.95), 0.03	0.82 (0.53, 1.27), 0.37
No	Ref	Ref
Ordering of questions about social risk screening and question about interest in assistance		
Answered questions about social risk factors first	Ref	Ref
Answered question about interest in assistance first	1.27 (0.90, 1.79), 0.18	1.56 (1.05, 2.31), 0.03
Social Risk Screening		
Number of risk factors[a]	3.22 (2.74, 3.78), <0.001	2.50 (1.96, 3.19), <0.001
Any prior social risk screening exposure in a health care setting in the past 12 months		
Yes	2.20 (1.24, 3.88), 0.007	1.41 (0.92, 2.15), 0.12
No	Ref	Ref
Any prior social risk assistance from health care setting in the past 12 months		
Yes	5.91 (3.70, 9.44), <0.001	1.76 (0.90, 3.45), 0.10
No	Ref	Ref
Any discomfort with questions in any screening domains		
Yes	4.02 (2.22, 7.28), <0.001	1.54 (0.70, 3.41), 0.29
No	Ref	Ref
Appropriateness of health care-based social risk screening		
Very/somewhat appropriate	1.18 (0.93, 1.50), 0.18	0.97 (0.65, 1.43), 0.87
Neither/very/somewhat inappropriate	Ref	Ref
Comfort with integrating social risk data in EHR		
Completely/somewhat comfortable	1.13 (0.74, 1.75), 0.57	1.27 (0.77, 2.11), 0.35
Neither/completely/ somewhat uncomfortable	Ref	Ref
Health care setting characteristics		
Primary Care	Ref	Ref
Emergency Department	1.49 (0.71, 3.13), 0.30	1.67 (0.95, 2.91), 0.07
Percentage patient population publicly-insured or uninsured		
<80%	Ref	Ref
≥80%	2.63 (1.35, 5.15), 0.005	1.66 (0.82, 3.38), 0.16

[a]Ordinal scale 0,1,2,3,4, 5

© 2020 De Marchis EH et al. *JAMA Network Open*.
